# 32^e^ Congrès national de la STPI 2^e^ Congrès francophone de Pathologie infectieuse et Microbiologie clinique 5 au 7 mai 2023, Hammamet, Tunisie

**DOI:** 10.48327/mtsi.v3i4.2023.432

**Published:** 2023-10-05

**Authors:** Adnene TOUMI, Hajer BEN BRAHIM, Aïda BERRICHE, Wissem HACHFI, Chakib MARRAKCHI, Lamia AMMARI, Nadia BEN LASFAR, Makram KOUBAA, Karim AOUN, Sourour NEJI, Rym BEN ABDALLAH, Meriem BOUCHEKOUA, Salma MHALLA, Habiba NAÏJA, Saba GARGOURI, Naïla HANNACHI, Lamia THABET, Basma MNIF, Wafa ACHOUR, Manel MARZOUK, Ilhem BOUTIBA, Jean-Philippe CHIPPAUX

**Keywords:** Antibiotiques, Antibiorésistance, Tuberculose, VIH, Candidoses, Zoonoses, Infections bactériennes, Infections virales, Infections fongiques, Infections parasitaire, Vaccinations, Tunisie, Maghreb, Afrique du Nord, Antibiotics, Antibiotic resistance, Tuberculosis, HIV, Candidiasis, Zoonosis, Bacterial infections, Viral infections, Fungal infections, Parasitic infections, Vaccinations, Tunisia, Maghreb, Northern Africa

 

