# Supplementary material for: 32nd National STPI Congress 2nd French-speaking Congress of Infectious Pathology and Clinical Microbiology 5 to 7 May 2023, Hammamet, Tunisia
Source: Med Trop Sante Int. 2023 Oct 5;3(4):mtsi.v3i4.2023.432. [Article in French] doi: 10.48327/mtsi.v3i4.2023.432 (PMC10879895; doi:10.48327/mtsi.v3i4.2023.432)
Supplement: Related texts

# Research article: Éditorial

**Published:** 2023-10-05

**Authors:** Karim AOUN

**Affiliations:** Professeur de Parasitologie à la Faculté de médecine de Tunis et à l'Institut Pasteur de Tunis. Institut Pasteur, 13 place Pasteur, BP 74, 1002 Tunis, Tunisie

Les pathologies infectieuses reviennent au-devant des actualités médicales et sociales, bien que beaucoup leur aient prédit un déclin programmé suite au développement de la vaccination, des chimiothérapies anti-microbes et des mesures adaptées d'hygiène, de prévention et de contrôle. En effet, nous avons assisté ces dernières années à la pandémie de Covid-19, à des épidémies de grippe graves et répétées, à l’émergence et à l'extension géographique de plusieurs maladies vectorielles (dengue, West Nile, leishmaniose cutanée), à une recrudescence de la tuberculose et du VIH et à l'aggravation du problème de l'antibiorésistance. Certaines épidémies, particulièrement celle due au Covid-19, ont révélé des insuffisances dans la prise en charge des patients (lits d'hospitalisation, cadres médicaux et paramédicaux spécialisés) et dans l'approvisionnement en vaccins, traitements (antibiotiques) et dispositifs (masques, oxygène). Par ailleurs, les progrès technologiques ont amélioré le diagnostic biologique (PCR, spectrométrie, séquençage, tests rapides) et ouvert de nouvelles voies dans la vaccination (vaccin à ARNm), permettant des avancées significatives dans plusieurs volets de la prévention et de la prise en charge ainsi qu'un bénéfice au niveau du pronostic de nombreuses maladies.

Le 2^e^ Congrès francophone de Pathologie infectieuse et de Microbiologie clinique couplé au 32e Congrès annuel de la Société Tunisienne de Pathologie Infectieuse (STPI) a placé au centre de ses thématiques plusieurs des aspects sus-cités. Il leur a été consacré des sessions spécifiques (virus émergents et ré-émergents, bon usage des antibiotiques, antibiorésistance, maladies tropicales négligées) au cours desquelles d’éminents experts et spécialistes ont été conviés pour en parler et exposer leurs expériences. La multidisciplinarité a été privilégiée, permettant un abord global des différents sujets traités. Ainsi, ont été réunis cliniciens, biologistes, vétérinaires, pharmaciens et chercheurs de 5 sociétés savantes partenaires investies dans le domaine de l'infectiologie. Les débats ont permis des échanges enrichissants et ne pourront qu'optimiser et renforcer les activités et les actions. L'utilisation du français comme langue officielle de l’évènement a représenté un avantage indéniable, particulièrement pour les jeunes praticiens des pays francophones de part et d'autre de la Méditerranée.

Le succès de cet évènement encourage fortement le renouvellement de telles expériences avec un partenariat entre plusieurs sociétés francophones et nous nous projetons déjà avec enthousiasme vers le congrès « Santé en Méditerranée » qui sera organisé les 22, 23 et 24 mai 2024 à Saint-Raphaël, en France, par la Société Francophone de Médecine Tropicale et Santé Internationale (SFMTSI) avec une large liste de partenaires régionaux dont la STPI et la Société de Pathologie Infectieuse de Langue Française (SPILF).

## La STPI

La Société Tunisienne de Pathologie Infectieuse (STPI) est une société savante scientifique créée en 1988. Elle est active dans les domaines des maladies infectieuses, de la microbiologie et de la parasitologie-mycologie. Elle est ouverte à tous les scientifiques engagés dans la santé publique et la recherche dans le domaine de l'infectiologie au sens large. Elle organise annuellement plusieurs manifestations scientifiques, notamment son congrès national annuel qui arrive en 2023 à sa 32^e^ édition.

Les principaux objectifs et missions de la STPI sont:

- Développer la qualité de prise en charge et de la prévention des maladies infectieuses en Tunisie;
- Assurer la formation des résidents en collaboration avec les collèges et sociétés savantes partenaires
- Participer au développement professionnel continu des médecins de première ligne;
- Participer à l’élaboration de consensus et à la réalisation d’études multicentriques;
- Renforcer la collaboration avec les autres sociétés savantes, à l’échelle nationale et internationale, particulièrement par l'organisation de cours de formation et d’évènements conjoints. La STPI a notamment développé des échanges réguliers avec la Société de Pathologie Infectieuse de Langue Française (SPILF) et la Société Algérienne d'Infectiologie (SAI). Un partenariat est en cours de mise en place avec la Société Francophone de Médecine Tropicale et Santé Internationale (SFMTSI);
- Participer aux activités de santé publique et aux programmes nationaux conduits par des structures nationales, particulièrement celles dépendant du Ministère de la Santé, ainsi que les projets soutenus par l'OMS;
- Promouvoir la recherche dans le domaine des maladies infectieuses.

La STPI expose ses activités dans un site web (www.infectiologie.tn) interactif qui est régulièrement actualisé et enrichi.

## L’évènement

Le 32^e^ congrès annuel de la STPI, couplé au 2^e^ Congrès francophone de Pathologie infectieuse et de Microbiologie clinique, est un évènement scientifique co-organisé par la Société Tunisienne de Pathologie Infectieuse (STPI) et la Société de Pathologie Infectieuse de Langue Française (SPILF), en partenariat avec la Société Francophone de Médecine Tropicale et Santé Internationale (SFMTSI) et la Société Algérienne d'Infectiologie (SAI). La manifestation a traité de l'infectiologie au sens large avec des sessions spécifiques concernant la bactériologie, la virologie, la parasitologie et la mycologie. Le congrès a privilégié la multidisciplinarité avec le développement de plusieurs aspects ayant trait plus particulièrement à l’épidémiologie, à la clinique, à la biologie, au traitement et au contrôle des maladies infectieuses. Il s'est déroulé les 5, 6 et 7 mai 2023 à Hammamet en Tunisie et a réuni plus de 300 participants de Tunisie, d'Algérie et de France. Le programme scientifique a comporté 4 ateliers interactifs de formation destinés aux jeunes résidents, 4 symposiums et 7 sessions thématiques. Vingt et une conférences ont été données par d’éminents spécialistes, et 14 cas cliniques interactifs et 488 travaux scientifiques ont également été présentés sous forme de communications affichées et d'e-posters.

**Figure 1:** Affiche du 32^e^ Congrès national de la STPI et du 2^e^ Congrès francophone de Pathologie infectieuse et Microbiologie clinique – 5 au 7 mai 2023, Hammamet, Tunisie Poster of the 32nd National STPI Congress – 2nd French-speaking Congress of Infectious Pathology and Clinical Microbiology – 5 to 7 May 2023, Hammamet, Tunisia

# Research article: F002 - Qu'en est-il du pneumocoque lors de cette dernière décennie?

**Published:** 2023-10-05

**Authors:** M. Marzouk, I. Bergaoui, Y. Maatouk, A. Belghouthi, S. Miri, I. Handous, N. Hannachi, J. Boukadida

**Affiliations:** Laboratoire de microbiologie, CHU Farhat Hached, Sousse, Tunisie

Outre son pouvoir pathogène varié et souvent grave, l’émergence de souches de *Streptococcus pneumoniae* résistantes aux antibiotiques est une cause majeure de morbi-mortalité en pathologie infectieuse.

## Objectif

Nous présentons les caractéristiques épidémiologiques et bactériologiques de ce pathogène durant les 10 dernières années dans la région de Sousse.

## Matériel et méthode

Il s'agit d'une étude rétrospective portant sur les souches non redondantes de *S. pneumoniae* isolées au laboratoire de microbiologie du CHU Farhat Hached de Sousse sur une période de 10 ans (2013-2022). L'identification bactérienne a été réalisée selon les techniques conventionnelles et l’étude de la sensibilité aux antibiotiques selon les recommandations du CASFM/EUCAST.

## Résultats

Un total de 265 souches de *S. pneumoniae* a été inclus dans notre étude, provenant principalement de la population masculine (sex-ratio H/F = 1,59). Le service le plus pourvoyeur d'infections à *S. pneumoniae* était la pédiatrie (23%) suivi de la chirurgie (13,2%) et des services de soins intensifs (10,9%). Les souches invasives (46%) provenaient essentiellement de LCR (25,6%) et d'hémoculture (20,3%). La majorité des souches non invasives (54%) correspondait à des prélèvements d'origine respiratoire (23,8%). Concernant la résistance aux bêtalactamines, le pneumocoque de sensibilité diminuée à la pénicilline (PSDP) représentait près de la moitié de nos souches (52%). La résistance à la pénicilline G, à l'amoxicilline et au céfotaxime étaient respectivement de 48,3%, 29,6% et 7%. Les taux de résistance aux autres familles d'antibiotiques étaient variables: érythromycine (53,1%), lincomycine (41,8%), tétracycline (26,5%). Aucune résistance aux glycopeptides n'a été rapportée. Une résistance de haut niveau à la gentamicine a été détectée chez 5,4% des cas.

## Conclusion

Les infections à *S. pneumoniae* en milieu hospitalier étaient graves et invasives dans près de la moitié des cas, associées à l’émergence des souches de PSDP. D'où l'intérêt des campagnes de sensibilisation auprès des médecins prescripteurs afin de maintenir un bon usage des antibiotiques.

# Research article: F011 - Antimicrobial stewardship: impact sur la consommation d'antibiotiques

**Published:** 2023-10-05

**Authors:** F. Sellami, A. Ben Cheikh Brahim, N. Ben Fatma, A. Jebri, A. Ferjani, I. Boutiba, A. Abassi

**Affiliations:** Service de pharmacie, service d'anesthésie réanimation, service de microbiologie, Hôpital Charles Nicolle, Tunis

La résistance aux antibiotiques est un processus naturel mais accéléré par leur utilisation excessive. C'est une menace majeure pour la santé publique.

## Objectif

Il s'agit d'une étude rétrospective étudiant l'impact d'un programme de gestion des antimicrobiens [AMSP] sur la rationalisation de la consommation d'antibiotiques dans notre hôpital.

## Matériel et méthode

La consommation d'antibiotiques en Unité commune de dispensation (UCD) a été collectée du logiciel STKMED puis convertie en Dose définie journalière (DDJ). Les résultats ont été exprimés en nombre de DDJ pour 1000 jours d'hospitalisation (JH). Nous avons analysé la consommation des services chirurgicaux, médicaux et réanimation, un an avant la mise en place de l'AMSP (2019) et un an après (2021). Les antibiotiques concernés étaient: glycopeptides, carbapénèmes, tétracycline, linézolide, fosfomycine et colimycine. Nous avons éliminé ces deux dernières molécules pour cause de rupture de stock sur l'une des périodes.

## Résultats

Nous avons noté une réduction de la consommation de la plupart des familles étudiées. Pour les services médicaux, les carbapénèmes et les glycopeptides ont été les familles les plus consommées au cours des deux années d’étude. Nous sommes passés de 16,23 DDJ/1000 JH à 6,49 DDJ/1000 JH et de 20,64 DDJ/1000 JH à 6,47 DDJ/1000 JH respectivement pour les deux familles entre 2019 et 2021. Pour les prestations chirurgicales, on note une diminution de la consommation d'ertapénem [4,57 DDJ/1000 JH à 1,08 DDJ/1000 JH] et d'imipénem+cilastatine [47,67 DDJ/1000 JH à 45,3 DDJ/1000 JH]. Concernant le service de réanimation, on note une diminution de la consommation d'antibiotiques de toutes les familles. Pour les glycopeptides, la consommation est passée de 294,57 DDJ/1000 JH en 2019 à 217,56 DDJ/1000 JH en 2021 [-26,14%] avec une augmentation de la consommation de teicoplanine contre une diminution de la consommation de vancomycine.

## Conclusion

Le suivi de la consommation d'antibiotiques est l'un des premiers indicateurs de la rationalisation des prescriptions. Le pharmacien hospitalier est à la croisée des chemins entre la rationalisation de la consommation d'antibiotiques et l'assurance d'une prise en charge adéquate.

# Research article: F013 - Impact de la réorganisation de l'activité de l’équipe mobile d'infectiologie

**Published:** 2023-10-05

**Authors:** M. Krifa, S. Kahloun, M. Ben Selma, M. Garma, M. Ben Ticha, M. Abid, F. Bellazreg, A. Letaief, W. Hachfi

**Affiliations:** Service des maladies infectieuses, CHU Farhat Hached, Sousse, Tunisie

Instaurée en 2011, l’Équipe mobile (EM) des maladies infectieuses du CHU Farhat Hached de Sousse a vu ses activités s’élargir pour répondre aux besoins des médecins de la région. Depuis octobre 2021, une réorganisation de notre activité a été conçue par la mise en place d'un numéro de téléphone mobile (TM) à la disposition d'un infectiologue référent qui assure une permanence téléphonique tous les jours.

## Matériel et méthode

L'activité de l'EM a été comparée entre deux périodes allant de 2016 à 2020 sans TM (= P1) et la période allant d'octobre à décembre 2022 avec TM ainsi que l’élaboration d'une nouvelle fiche d'activité en 2022 (= P2). Celle-ci est remplie et saisie en rétrospectif sur une base de données informatisée SPSS.26. Les interventions effectuées aux urgences et à la consultation externe n'ont pas été incluses.

## Résultats

Durant la P1, une moyenne de 157 avis par trimestre a été notée contre 470 avis durant P2 (soit 3 fois plus). Cette élévation intéresse essentiellement le CHU Sahloul allant de 30 (19%) en P1 à 106 (35%) en P2. Parmi les services qui nous ont sollicités, l'endocrinologie vient en premier lieu (10%) suivi de l'ORL (7%) dans la P1, alors que dans la P2, le service d'ORL nous a le plus sollicités (9,2%). Les interventions ont été faites par téléphone dans 69 cas (44%) dans la P1 contre 164 cas (54%) dans la P2, soit 2 fois plus. Un déplacement au lit du malade a été nécessaire dans 125 cas (80%) dans la P1 contre 116 cas (25%) dans la P2. La principale intervention de l'EM consistait en une aide au traitement dans 125 cas en P1 contre 224 cas en P2 (soit 1,7 fois plus). Aucune prescription d'antibiotiques n'a été proposée dans 13 cas (9%) dans la P1 contre 39 (12,7%) dans la P2. L'arrêt de l'antibiothérapie a été suggéré dans 11 cas (8%) en P1 contre 28 (9,2%) en P2.

## Conclusion

La mise à la disposition de l'EM d'un TM et la réorganisation de son activité ont facilité l'accès à un avis spécialisé. Le nombre d'avis médicaux intra et interhospitaliers a triplé. La demande d'aide au traitement a doublé, et notre avis a permis de réduire la prescription d'antibiotiques dans 9,2% des cas.

# Research article: F014 - Activité *in vitro* du ceftazidime/avibactam et du ceftolozane/tazobactam sur les *Pseudomonas aeruginosa* isolées au CTGB

**Published:** 2023-10-05

**Authors:** Z. Megdiche, B. Maamar, B. Ben Della, S. Dhraief, L. Thabet

**Affiliations:** Laboratoire de biologie médicale, Centre de traumatologie et des grands brûlés (CTGB), Université Tunis El Manar, Faculté de médecine de Tunis, UR22SP03

Ceftazidime-avibactam (CZA) et ceftolozane-tazobactam (C/T) peuvent avoir un rôle potentiellement important dans la prise en charge des infections à *P. aeruginosa,* particulièrement celles causées par les souches multirésistantes et ultrarésistantes aux antibiotiques.

## Objectif

Évaluer l'efficacité *in vitro* des associations CZA et C/T sur les souches de *P. aeruginosa* isolées au Centre de traumatologie et des grands brûlés (CTGB).

## Matériel et méthode

Notre étude est rétrospective descriptive, menée au laboratoire de biologie médicale du CTGB pendant 7 mois (août 2022-février 2023). Nous avons inclus toutes les souches de *P. aeruginosa* isolées à partir des prélèvements à visée diagnostique. L'identification bactérienne était faite selon les méthodes conventionnelles et l’étude de la sensibilité aux antibiotiques selon les normes du CASFM 2022. La recherche et le typage moléculaires de carbapénémases ont été réalisés par une PCR GeneXpert’ (Cepheid) par le kitXpert’ Carba-R détectant les principaux gènes codant pour les carbapénémases (blaVIM, blaNDM, blaIMP, blaOxa48 et blaKPC).

## Résultats

Durant la période d’étude, 161 souches non redondantes de *P. aeruginosa* ont été isolées, essentiellement des prélèvements provenant des services de réanimation des brûlés (45%) et d'anesthésie-réanimation (19%). La résistance à CZA et C/T était de 42,2% et 47,8% des souches respectivement, occupant ainsi la 2^e^ et la 3^e^ place des bêtalactamines les plus actives après l'aztréonam (AZT). Parmi les 81 souches de *P. aeruginosa* résistantes à la ceftazidime (CAZ-R), la résistance passait à 85% et 96,6% au CZA et C/T respectivement contre 97,5% pour l'imipénem (IMP), le méropénem (MER) et l'aztréonam. Les 86 souches résistantes aux carbapénèmes avaient une résistance de 71% et 81,4% au CZA et C/T respectivement, après l'aztréonam qui avait un taux de résistance de 41,9%. Parmi les souches CZA résistantes (n = 68), 39,7% étaient des souches productrices de métallo-bêta-lactamases de type VIM.

## Conclusion

Dans notre étude, CZA représente la bêta-lactamine la plus active contre les souches de *P. aeruginosa* CAZ-R, et la deuxième la plus active sur toutes les souches de *P. aeruginosa* ainsi que celles productrices de carbapénémases. Son usage doit cependant être réservé, après documentation, aux souches multirésistantes.

# Research article: F018 - Apport de la Mismatch Amplification Mutation Assay-PCR dans la détection des mutations gyrA et parC chez *Escherichia coli* au Centre national de greffe de moelle osseuse

**Published:** 2023-10-05

**Authors:** K. Bouhalouene, Y. Chebbi, A. Raddaoui, S. Frigui, W. Achour

**Affiliations:** 1 Service des laboratoires, Centre national de greffe de moelle osseuse, 1006 Tunis, Tunisie; 2 Université Tunis El Manar, Faculté de médecine de Tunis, LR18ES39, 1006 Tunis

La résistance aux fluoroquinolones (FQ) est principalement due à des mutations ponctuelles des gènes gyrA et parC dans les régions déterminant la résistance aux quinolones (QRDR). La technique de séquençage permet d'identifier ces mutations, mais son coût élevé et sa lourdeur ont conduit au développement d'autres techniques moléculaires pour détecter les mutations les plus fréquentes.

## Objectif

Déterminer la fréquence des mutations des gènes gyrA et parC par Mismatch Amplification Mutation Assay-PCR (MAMA-PCR) chez les souches d’*E. coli* résistantes aux FQ (EC-RQ) isolées au Centre national de greffe de moelle osseuse (CNGMO).

## Matériel et méthode

Une étude rétrospective incluant les souches non répétitives d'EC-RQ responsables de colonisation ou d'infection chez les patients consultants ou hospitalisés au CNGMO a été menée entre janvier 2019 et décembre 2021. L’étude de la sensibilité aux antibiotiques a été réalisée selon les recommandations du CASFM 2019. Les mutations dans la région QRDR de gyrA 83, gyrA 87, parC 80 et parC 84 ont été détectées par MAMA-PCR. Les gènes de résistances plasmidiques aux fluoroquinolones (AAC-6’-ib-cr, QnrS, QnrA etQnrB) ont été détectés par amplification PCR.

## Résultats

Au total, 123 souches d'EC-RQ ont été collectées, essentiellement dans l'unité de greffe adulte (43,9%). Elles étaient responsables principalement de colonisation digestive (n = 114; 92,7%). Les taux de résistance aux FQ chez ces souches étaient de 80,5% à la ciprofloxacine, de 90,1% à la norfloxacine et de 95,6% à l'ofloxacine. Les taux de résistance associés aux antibiotiques étaient de 91,5% à l'amoxicilline-acide clavulanique, de 97,4% à la ceftazidime, de 17,4% à l'ertapénem, de 9,9% à l'imipénem, de 19,67% à l'amikacine, de 70,7% à la gentamicine et de 76,3% au cotrimoxazole. Toutes les souches étaient porteuses de la mutation de gyrA 83, 27,6% étaient porteuses de la mutation parC 80, 13% de la mutation parC 84 et 7,3% de la mutation gyrA 87. Les gènes plasmidiques AAC-6’-ib-cr, QnrB et QnrS étaient retrouvés chez 52%, 17% et 13,9% des souches, respectivement. Les souches résistantes à la ciprofloxacine avaient essentiellement les génotypes: gyrA 83 (26%), gyrA 83+AAC6’-ib-cr (14%) et gyrA 83+parC 80+AAC-6’ib-cr (10,5%).

## Conclusion

Nous avons observé une fréquence faible des génotypes associant deux mutations gyrA parmi les souches résistantes à la ciprofloxacine au CNGMO. Ceci serait lié au faible nombre de mutations ciblées par la MAMA-PCR. La sensibilité de cette technique étant dépendante de l’épidémiologie locale de la résistance (fréquence des gènes ciblés).

# Research article: F019 - Quanti F ERO N®-TB Gold-Plus: intérêt de la mesure de la réponse CD8+ dans la détermination du statut de la tuberculose

**Published:** 2023-10-05

**Authors:** M. Mejri, A. Sfaxi, E. Drissi, N. Zmerli, N. Ghrairi, S. Yalaoui

**Affiliations:** Service de biologie, Hôpital Abderrahmen Mami, Ariana, Tunisie

Le QuantiFERON Gold-Plus (QFT-Plus) permet de quantifier la réponse des lymphocytes CD8+. La réponse CD8 serait plus importante chez les patients atteints de tuberculose maladie par rapport à ceux qui ont une tuberculose infection latente.

## Objectif

Ce travail avait pour but de déterminer le seuil de la réponse CD8+ qui permet de faire la distinction entre tuberculose active et latente.

## Matériel et méthode

Il s'agit d'une étude rétrospective menée de janvier 2017 à janvier 2023 sur les demandes de test QFT reçues au laboratoire de biologie de l'hôpital Abderrahmen Mami de l'Ariana.

## Résultats

Nous avons colligé 409 demandes de test QuantiFERON. L’âge moyen des patients était de 42 ans avec un sex-ratio de 0,8. Les principaux motifs de demande de test au QFT étaient les suivants: recherche d'une tuberculose latente dans le cadre d'un bilan pré-thérapeutique dans 234/409 cas soit 57%, recherche de tuberculose extrapulmonaire dans 140/409 cas soit 34%, recherche de cas contact dans 35/409 cas soit 9%. Le test au QFT était positif chez 102/409 patients. Le diagnostic de tuberculose active était retenu chez 45/102 malades. Le diagnostic de tuberculose latente était retenu chez 57/102 patients. La moyenne de la réponse CD8 (TB2-TB1) était de 0,65 UI/mL chez les malades qui avaient une tuberculose active et de 0,030 UI/mL chez les malades ayant une tuberculose latente. La comparaison de la réponse CD8+ entre les patients qui avaient une tuberculose active *versus* tuberculose latente a montré qu'une valeur seuil de 0,3 UI/mL était en faveur d'une tuberculose active (p < 0,01).

## Conclusion

La réponse CD8 était plus élevée chez les patients qui avaient une tuberculose active par rapport aux patients qui avaient une tuberculose latente. Une réponse CD8 (TB1-TB2) supérieure à 0,3 UI/mL était en faveur d'une tuberculose latente.

# Research article: F020 - Dix ans de surveillance épidémiologique des infections à pneumocoque dans la région de Sfax (2012-2021)

**Published:** 2023-10-05

**Authors:** O. Gargouri, N. Ben Ayed, S. Ktari, S. Bougharriou, S. Mezghani, O. Gargouri, B. Mnif, F. Mahjoubi, A. Hammami

**Affiliations:** Laboratoire de microbiologie, CHU Habib Bourguiba, Sfax, Tunisie

Les infections à *Streptococcus pneumoniae* constituent un problème de santé publique. La surveillance de la résistance aux antibiotiques et de la distribution des sérotypes permet de fournir des informations importantes pour guider la prescription d'antibiotiques et les programmes de vaccination.

## Objectif

Déterminer la distribution des sérotypes et étudier la résistance aux antibiotiques des souches de pneumocoque isolées à Sfax entre 2012 et 2021.

## Matériel et méthode

Il s'agit d'une étude rétrospective incluant toutes les souches de *S. pneumoniae* isolées au laboratoire de microbiologie du CHU Habib Bourguiba de Sfax entre 2012 et 2021. L’étude de la sensibilité aux antibiotiques a été effectuée selon les recommandations du Comité de l'antibiogramme de la Société française de microbiologie (CASFM) de l'année correspondante. Le sérotypage des souches a été réalisé par des réactions de PCR multiplex et par agglutination à l'aide de sérums spécifiques (ImmuLex™ Pneumotest).

## Résultats

Durant la période d’étude, 637 souches non redondantes de *S. pneumoniae* ont été isolées. L’âge moyen des patients était de 34 ans (1 jour – 92 ans). 26,4% des souches étaient invasives. Parmi les souches isolées, 413 (66,6%) avaient une sensibilité diminuée à la pénicilline (PSDP) avec un taux de résistance (R+I) à l'amoxicilline et au céfotaxime respectivement de 50,2% et 12,8%. Les taux de résistance (R+I) étaient également élevés pour l’érythromycine-clindamycine (69,3% – 59,9%), la tétracycline (46%) et le triméthoprime/sulfaméthoxazole (27,4%). Ces taux de résistance ont été fluctuants au cours des années sans tendance évolutive significative. 557 souches ont été sérotypées. Les sérotypes les plus fréquents étaient le 19F (17,6%) suivi par les sérotypes 14 (15,1%), 19A (9,5%), 3 (9,2%), 23F (6,6%) et 6B (6,6%). La couverture vaccinale était de 55,6% pour le VPC10 et 78,8% pour le VPC13. Une augmentation significative de la fréquence a été notée pour le sérotype 19A (rho = 0,855; p = 0,002).

## Conclusion

Les taux alarmants de résistance aux antibiotiques et l'augmentation de la fréquence des sérotypes non inclus dans le VPC10, vaccin introduit dans notre programme national d'immunisation depuis 2019, nécessitent une surveillance particulière.

# Research article: F021 - Vingt ans de surveillance épidémiologique des shigelloses à Sfax, Tunisie

**Published:** 2023-10-05

**Authors:** F. Maalej, M. Guetat, F. Gargouri, S. Ktari, N. Ben Ayed, F. Mahjoubi, S. Mezghani, A. Hammami

**Affiliations:** Laboratoire de microbiologie, CHU Habib Bourguiba, Sfax, Tunisie

La shigellose est une maladie diarrhéique, responsable d’épidémies à travers le monde. Cette infection pose de plus en plus de problèmes thérapeutiques étant donné la diffusion mondiale de souches de *S. sonnei* multirésistantes aux antibiotiques depuis 2008 dans plusieurs pays d'Europe. L'objectif de l’étude a été de suivre le profil épidémiologique et bactériologique des shigelloses dans la région de Sfax à partir de 2003 et de décrire les particularités de l’épidémie à *S. sonnei* en 2022. Il s'est agi d'une étude rétrospective des souches de shigelles isolées au laboratoire de microbiologie du CHU Habib Bourguiba de Sfax pendant 20 ans (2003 à 2022). L'identification a été faite par Vitek2 (BioMérieux”). Le sérotype a été déterminé par agglutination (Biorad). L’étude de la sensibilité aux antibiotiques a été déterminée par la méthode de diffusion des disques selon les normes du CASFM. Les CMI (E-test) de l'amoxicilline-acide clavulanique (AMC) et de la Ciprofloxacine ont été déterminées pour les souches résistantes aux céphalosporines de 3^e^ génération (C3G) et à la péfloxacine respectivement. L’étude de la clonalité des souches a été faite par électrophorèse en champ pulsé. Durant la période d’étude, 113 souches de shigelles ont été collectées dont 59 (52,2%) de 2003 à 2021: moyenne annuelle de 3 souches (min = 0, max = 16) et 54 (47,8%) en 2022.

La majorité de ces souches était isolée à partir des coprocultures (92,9%) suivie par les urines (4,4%) et les hémocultures (2,7%). De 2003 à 2021, les principales espèces isolées étaient *S. flexneri* (52,5%) et *S. sonnei* (30,5%) suivies par *S. boydii* (11,9%), *S. dysenteriae* (1,7%) et *Shigella* sp. (3,4%). Toutes ces souches étaient sensibles aux C3G, aux aminosides et aux fluoroquinolones; 15,3% étaient résistantes à l'AMC et 35,6% au cotrimoxazole. À partir de juillet 2022, une flambée de diarrhée invasive à *S. sonnei* chez des enfants âgés de 9 mois à 16 ans a été mise en évidence. En effet, en 2022, 53 souches de *S. sonnei* et une souche de *S. flexneri* ont été isolées. Toutes ces souches de *S. sonnei* étaient résistantes aux C3G par production de bêta-lactamase à spectre élargi, au cotrimoxazole et de sensibilité diminuée aux fluoroquinolones (CMI de la ciprofloxacine de 0,25 à 1 mg/L). Par ailleurs, toutes ces souches étaient sensibles aux aminosides, aux carbapénèmes, à la fosfomycine et aux furanes. Une seule souche était résistante à l'AMC (CMI = 12 mg/L). La souche de *S. flexneri* isolée en 2022 était de phénotype sauvage. L’étude de la clonalité faite sur un échantillon de souches de *S. sonnei* isolées en 2022 montrait la présence de pulsotypes similaires suggérant leur appartenance à un seul clone.

L’épidémie, dans la région de Sfax, de *S. sonnei* multirésistante aux antibiotiques constitue un problème majeur de santé publique. L'application des mesures d'hygiène est nécessaire pour la contrôler. Une étude de l'activité de l'azithromycine est nécessaire afin de guider l'antibiothérapie probabiliste.

# Research article: F023 - Étude de l'activité de la ceftazidime-avibactam, ceftolozane-tazobactam et céfidérocol vis-à-vis des souches de *Pseudomonas aeruginosa* résistantes à la ceftazidime à Sfax

**Published:** 2023-10-05

**Authors:** S. Rebaii, O. Gargouri, R. Kharrat, N. Ben Mansour, N. Ben Ayed, F. Mahjoubi, A. Hammami, B. Mnif

**Affiliations:** Laboratoire de microbiologie, CHU Habib Bourguiba, Sfax, Tunisie

Les infections dues à *Pseudomonas aeruginosa* résistant à la ceftazidime (PA-CAZ) posent un sérieux défi thérapeutique. Les nouveaux antibiotiques constituent une option thérapeutique prometteuse. L'objectif a été d’étudier l'activité des nouveaux antibiotiques, la ceftazidime-avibactam, la ceftolozane-tazobactam et le céfidérocol sur des souches de PA-CAZ isolées à Sfax. L’étude rétrospective a inclus 74 souches de PA-CAZ isolées des hémocultures reçues entre 2015 et 2022 au laboratoire de microbiologie du CHU Habib Bourguiba de Sfax. L’étude de la sensibilité aux antibiotiques a été réalisée par la méthode de diffusion des disques selon les recommandations du CASFM. La CMI de la colistine a été déterminée par la méthode de microdilution. Les gènes codant les métallobêta-lactamases ont été recherchés par PCR.

Parmi les 74 souches de PA-CAZ, 3,1% étaient sensibles à l'imipénem, 7,8% à l'amikacine, 40% à la ciprofloxacine et 66,2% à la colistine; 51,3% de ces souches étaient productrices de la métallo-bêta-lactamase de type VIM (MBL-VIM) dont 94,3% étaient résistantes à l'amikacine et 35,3% à la ciprofloxacine; 25 souches (33,8%) parmi les 74 souches de PA-CAZ, étaient sensibles à la ceftazidime-avibactam, 43 (58,1%) étaient résistantes et 6 (8,1%) étaient catégorisées ZIT (zone d'incertitude technique). La majorité des souches résistantes à la CAZ (83,3%) étaient productrices de la MBLVIM; 22 souches (29,7%) étaient sensibles au ceftolozane-tazobactam et 52 (70,3%) résistantes dont 37 (71,1%) étaient productrices de la MBL-VIM; 68 souches (91,9%) étaient sensibles au céfidérocol; une souche (1,4%) était résistante et 5 souches (6,7%) étaient catégorisées en ZIT.

Notre étude a mis en évidence une bonne activité de la ceftazidime-avibactam et du ceftolozane-tazobactam sur les souches de PA-CAZ à Sfax ainsi qu'une excellente activité du céfidérocol, d'où l'intérêt de les tester et de les introduire comme alternatives thérapeutiques pour les infections à PA-CAZ en Tunisie.

# Research article: F024 - Étude de la sensibilité à la ceftazidime-avibactam des entérobactéries isolées d'infections urinaires et résistantes aux carbapénèmes

**Published:** 2023-10-05

**Authors:** K. Cherif, S. Boughattas, F. Azzouzi, L. Tilouche, S. Naija, Y. Benlammine, S. Ketata, A. Trabelsi

**Affiliations:** Service de microbiologie du CHU Sahloul, Sousse, Tunisie

L’émergence des entérobactéries productrices de carbapénémases (EPC) constitue un problème majeur dans la prise en charge des patients présentant des infections urinaires (IU) avec une augmentation du taux de morbi-mortalité. L'objectif de cette étude était de rechercher et de typer les carbapénémases chez les souches d'entérobactéries isolées d'infections urinaires et d’étudier la sensibilité de celles-ci à la ceftazidime-avibactam (CZA). Il s'agissait d'une étude rétrospective analytique menée au service de microbiologie du CHU Sahloul de Sousse sur une période de 2 ans (2020-2021) et portant sur les souches d'entérobactéries isolées d'infections urinaires et résistantes à l'ertapénem. L’étude de la sensibilité aux antibiotiques a été réalisée selon les recommandations du CASFM/EUCAST 2020. La recherche de carbapénémase a été réalisée par une méthode phénotypique basée sur les tests d'inhibition sur milieu gélosé à l'aide du kit ROSCO Diagnostica”. L’étude de la sensibilité à la CZA a été réalisée pour toutes les souches non productrices de métallo-bêta-lactamases (MBL) par la technique E-test.

Quarante-six souches d'entérobactéries résistantes à l'ertapénem ont été colligées, représentées principalement par *Klebsiella pneumoniae* (KP, 70%) suivie d’*Escherichia coli* (17%) puis d’*Enterobacter* spp. (13%). Parmi ces souches, 35 (76%) étaient des EPC. Au sein des EPC, 19 souches (54,3%) étaient productrices de MBL et 18 (51,4%) étaient productrices d'OXA-48. L'association des deux enzymes a été trouvée dans deux souches. Parmi les EPC, 33% des souches étaient isolées chez des patients du service de chirurgie neurologique suivi par le service de néphrologie (20%). Toutes les souches d'entérobactéries résistantes à l'ertapénem non productrices de MBL (n = 27; 58,7%) étaient sensibles au CZA.

La ceftazidime-avibactam pourrait être une alternative dans le traitement des IU à EPC. L’émergence de souches productrices de MBL nous prive de cette molécule. La rationalisation de l'usage des antibiotiques et l'application des mesures d'hygiène s'imposent afin de préserver l'arsenal thérapeutique.

# Research article: F025 - Caractérisation moléculaire des *Escherichia coli* producteurs de BLSE responsables d'infections communautaires et nosocomiales en Tunisie

**Published:** 2023-10-05

**Authors:** N. Sallem, N. Ben Mansour, A. Hammami, B. Mnif

**Affiliations:** Laboratoire de microbiologie, CHU Habib Bourguiba, Sfax, Tunisie

Les *Escherichia coli* producteurs de BLSE (E-BLSE) constituent un problème de santé public mondial tant dans le milieu hospitalier que communautaire. L'objectif était de comparer les caractéristiques microbiologiques des *Escherichia coli* producteurs de BLSE (E-BLSE) isolés d'infections communautaires et nosocomiales en Tunisie. Cette étude rétrospective a inclus toutes les souches d'EBLSE isolées au laboratoire de microbiologie de l'hôpital Habib Bourguiba de Sfax entre 2000 et 2019 à partir d'infections nosocomiales confirmées. D'autre part, une collection de 107 souches d'E-BLSE isolées d'infections communautaires (E-BLSECom) entre 2011 et 2016 a été réalisée après un interrogatoire excluant toute relation de l'infection avec les soins. Le type de BLSE a été confirmé par PCR-séquençage. Les groupes phylogénétiques, 28 facteurs de virulence et 8 systèmes de toxines et antitoxines ont été déterminés par PCR.

Quatre cent quatre-vingt-huit E-BLSE-Noso ont été recensées à l'hôpital Habib Bourguiba de Sfax avec une augmentation significative de l'incidence passant de 0,08 à 0,32 cas/1000 journées d'hospitalisation entre 2000 et 2019. Ces souches ont été isolées majoritairement d'urines (53,6%) et d'hémocultures (10,7%). Parmi les 371 E-BLSE-Noso conservées et étudiées, 380 BLSE ont été détectées dont 92% étaient des CTX-M avec 251 CTX-M-15, 54 CTX-M-27, 24 CTXM-14 et 21 CTX-M-1. De même, pour les 107 E-BLSE-Com, cellesci étaient isolées quasi exclusivement au cours d'infections urinaires (93%) et produisaient majoritairement des CTX-M (94%) avec 74 CTX-M-15, 20 CTX-M-27, 8 CTXM14 et 4 CTX-M-1. Néanmoins, la proportion des souches appartenant au clone B2-ST131 était significativement plus élevée pour les E-BLSE-Com (63,6%) que pour les E-BLSENoso (42,3%, p = 0,002). Le clone ST131 C1M27 était significativement plus associé aux souches nosocomiales de même que la coproduction de carbapénémase. Les souches E-BLSE-Com étaient significativement plus riches en facteurs de virulence (FV) aussi bien pour les souches ST131 (moyenne de FV par souche 16,6 vs 15,1; p = 0,0008) que les non-ST131 (moyenne de FV par souche 8,2% vs 7,2%).

Notre étude a mis en évidence des caractéristiques microbiologiques différentes entre les E-BLSE-Noso et les E-BLSE-Com, ces dernières s'avérant plus virulentes et plus associées au clone ST131 mais moins résistantes aux antibiotiques que les E-BLSE-Noso.

# Research article: F027 - Etude phénotypique et génotypique des entérobactéries productrices des ß-lactamases à spectre étendu isolées à l'hôpital Aziza Othmana

**Published:** 2023-10-05

**Authors:** S. Khedher, H. Hannachi, W. Kalai, M. Hamdoun, S. Hammoudi, S. Ferjani, R. Bounawara, S. Hamdi, I. Boutiba, O. Bahri

**Affiliations:** 1 Laboratoire de microbiologie-biochimie, Hôpital Aziza Othmana, Tunis, Tunisie; 2 Laboratoire de microbiologie, Hôpital Charles Nicolle, Tunis

Avec l'utilisation de plus en plus élargie des antibiotiques, l'antibiorésistance est devenue un problème majeur de santé publique. L'objectif de cette étude était de déterminer le profil phénotypique et génotypique des souches d entérobactéries productrices des β-lactamases à spectre étendu (E-BLSE) isolées à l'hôpital Aziza Othmana de Tunis. Cette étude rétrospective descriptive s'est étalée sur une période allant du 1^er^ janvier 2019 au 31 juillet 2022. La détermination de la sensibilité aux antibiotiques a été réalisée selon les recommandations internationales. La détection des gènes codant pour les BLSE (blaTEM, blaSHV, blaOXA, blaGES, blaVEB, blaPER, et blaCTX-M avec ses sous-groupes) a été effectuée par *polymerase chain reaction* (PCR) après extraction de l'ADN bactérien par lyse thermique. Un total de 96 souches d'E-BLSE a été étudié. Elles ont été isolées chez des patients hospitalisés essentiellement en hématologie (84,4%) et appartenant aux tranches d’âge 25-35 ans (21%) et 55-65 ans (21%). Il s'agissait essentiellement de patientes de sexe féminin (59,4%). Ces souches provenaient principalement d'un portage digestif (60,4%). *Escherichia coli* était l'espèce la plus représentée (61,5%), suivie de *Klebsiella pneumoniae* (37,5%). La majorité des souches d'E-BLSE avait une résistance à l'association sulfaméthoxazole/triméthoprime (71,1%), à la ciprofloxacine (56,3%) et à la nitrofurantoïne (33,3%). Concernant les aminosides, les taux de résistance étaient de 37,9% pour la tobramycine, 28,5% pour la gentamicine et 10,4% pour l'amikacine. La sensibilité à la tigécycline et à la fosfomycine, par contre, était sauvegardée; le taux de résistance était d'uniquement 19,1% et 7,9% respectivement. La détermination génotypique montrait une prédominance des blaCTX-M-1 (76,3%). Des souches blaTEM étaient retrouvées dans 44% des cas. Les fréquences de blaOXA et blaSHV étaient équivalentes (dans 31% des cas); blaCTX-M-9 était le profil de résistance le moins fréquent (5,2%).

Cette étude confirme l’évolution des β-lactamases bactériennes vers la diversification et l’élargissement de leur spectre d'activité, d'où l'importance de l'instauration d'une politique de surveillance de la résistance aux antibiotiques, particulièrement chez les sujets à haut risque infectieux.

# Research article: F040 - Apport du GeneXpert^®^ MTB/RIF Ultra dans le diagnostic de la tuberculose extrapulmonaire

**Published:** 2023-10-05

**Authors:** E. Moalla, Y. Chelbi, A. Ferjani, L. Kanzari, I. Boutiba-Ben Boubaker

**Affiliations:** 1 Laboratoire de microbiologie, Hôpital Charles Nicolle, 1006 Tunis, Tunisie; 2 Laboratoire de recherche « Résistance aux antimicrobiens » LR99ES09, Faculté de médecine de Tunis, Université Tunis El Manar, 1007 Tunis

Les formes extrapulmonaires de la tuberculose sont actuellement de plus en plus fréquentes en Tunisie. Leur diagnostic microbiologique est difficile vu leur caractère pauci-bacillaire, mais amélioré par les techniques de biologie moléculaire. L'objectif de cette étude était de décrire l'apport du GeneXpert” MTB/RIF Ultra dans le diagnostic de la tuberculose extrapulmonaire. Il s'agissait d'une étude rétrospective sur 3 ans (janvier 2020 – décembre 2022), menée au laboratoire de microbiologie de l'Hôpital Charles Nicolle. Elle concernait tous les prélèvements extrapulmonaires adressés pour recherche de mycobactéries du complexe *tuberculosis* à la fois par méthodes conventionnelles et par GeneXpert” MTB/RIF Ultra. L'examen direct à la recherche de bacilles acido-alcoolo-résistants (BAAR) était basé sur une coloration à l'auramine confirmée par la coloration de Ziehl-Nelson. Les prélèvements étaient ensemencés sur milieu de Lowenstein-Jensen pendant 60 jours. Une coloration de Ziehl-Nelson était pratiquée pour les cultures positives. En cas de présence des BAAR, le test immunochromatographique Bioline” TB AgMPT64 était réalisé pour identifier les mycobactéries du complexe *tuberculosis*.

Au total, 82 prélèvements étaient reçus, représentés essentiellement par les prélèvements ganglionnaires (n = 20; 24%) et les ponctions pleurales (n =10; 12%). L'examen direct était positif sur un seul prélèvement. Le test de GeneXpert” MTB/RIF Ultra était positif, douteux ou négatif pour respectivement 31, 7 et 44 prélèvements. Le test était positif pour 14/20 prélèvements ganglionnaires, 2/10 ponctions pleurales, 5/11 ponctions lombaires, 1/8 ponctions d'ascite, 1/1 ponction articulaire, 1/9 prélèvements d'urines, 1/6 biopsies cutanées, 4/9 biopsies osseuses et 2/6 prélèvements de pus. Tous les prélèvements avec GeneXpert négatif avaient une culture et un examen direct négatifs. Parmi les 38 prélèvements positifs et douteux en GeneXpert, un seul prélèvement ganglionnaire était positif à l'examen direct. Celui-ci avait aussi une culture positive à *Mycobacterium tuberculosis.* Aucune résistance à la rifampicine n’était détectée dans cette série.

La réalisation du GeneXpert” MTB/RIF Ultra présente un excellent apport dans le cadre du diagnostic de la tuberculose extrapulmonaire et permet de rattraper les résultats négatifs de la culture.

# Research article: F041 - Prise en charge diagnostique et thérapeutique de la tuberculose ganglionnaire cervicale

**Published:** 2023-10-05

**Authors:** F. Hammami, M. Koubaa, K. Rekik, A. Chakroun, C. Marrakchi, F. Smaoui, M. Ben Jemaa

**Affiliations:** Service des maladies infectieuses et Unité de recherche de la tuberculose extrapulmonaire, CHU Hédi Chaker, Sfax, Tunisie

La tuberculose demeure un problème de santé publique mondial. La localisation ganglionnaire, dont la localisation cervicale, représente la forme la plus fréquente de la tuberculose extrapulmonaire. Elle pose encore un problème diagnostique et thérapeutique. L'objectif était d’étudier les caractéristiques cliniques, thérapeutiques et évolutives de la tuberculose ganglionnaire cervicale (TGC). Nous avons mené une étude rétrospective incluant tous les patients hospitalisés au service des maladies infectieuses pour une TGC entre 1995 et 2021.

Nous avons colligé 297 patients dont 207 étaient des femmes (69,7%). L’âge moyen était 37 ± 19 ans. Les patients âgés de 19 à 39 ans étaient la tranche d’âge la plus fréquente (41,4%). Au total, 173 patients avaient une origine rurale (58,6%). Des antécédents personnels (7,4% des cas) et familiaux (7,1%) de tuberculose étaient notés. Les signes d'imprégnation tuberculeuse incluaient une fièvre (44,1% des cas), une asthénie (44,4%), un amaigrissement (36,7%) et des sueurs nocturnes (32,1%). L'intradermoréaction à la tuberculine était positive dans 135 cas (45,5%). Il s'agissait d'une tuberculose multifocale dans 61 cas (20,5%). Une tuberculose abdominale (10,8% des cas), neuroméningée (3,7%), urogénitale (3,4%) et osseuse (3,4%) était associée. Une cytoponction (71,7% des cas) et une biopsie ganglionnaire (40%) étaient réalisées. Le diagnostic était confirmé par une preuve anatomopathologique dans 80,8% des cas et une preuve microbiologique dans 20,5% des cas. Dans le prélèvement ganglionnaire, la recherche de bacille de Koch à l'examen direct (6,4% des cas) et/ou après culture (3,7%) ou le test de biologie moléculaire (10,4%) était positive. Le traitement antituberculeux était prescrit dans sa forme combinée dans 183 cas (61,6% des cas). La durée moyenne du traitement était de 9 ± 4 mois. Des effets indésirables étaient notés dans 85 cas (28,6%). Ils s'agissaient d'une cytolyse hépatique (12,1% des cas), d'une intolérance digestive (7,1%), d'un prurit (4,4%), de paresthésie (3,7%) et d'une leucopénie (3,4%). L’évolution était favorable dans 263 cas (88,5%). Des complications (11,1% des cas), des séquelles (6,1%) et une rechute (4,4%) étaient notées. Trois patients étaient décédés (1% des cas).

Le diagnostic de la TGC doit être évoqué devant une tuméfaction cervicale associée ou non à des signes d'imprégnation tuberculeuse et à une intradermoréaction à la tuberculine positive. Une cytoponction ou, à défaut, une biopsie ganglionnaire permet de confirmer le diagnostic et de débuter précocement le traitement.

# Research article: F042 - Tuberculose pulmonaire: facteurs associés au retard de négativation des BK

**Published:** 2023-10-05

**Authors:** E. Zaara, R. Kaddoussi, W. Gharbi, L. Loued, A. Ben Saad, A. Ghourabi, A. Migaw, K. Felhi, S. Ksissa, S. Joober, S. Cheikh Mhamed, N. Fahem

**Affiliations:** Service de pneumologie, Hôpital Fattouma Bourguiba, Monastir, Tunisie

La tuberculose, bien traitée, évolue généralement vers la guérison. Cependant, un retard de la négativation des bacilles de Koch dans les prélèvements qui est défini par un délai dépassant les 2 mois peut être observé. Cela est lié à plusieurs facteurs, d'où l'intérêt de notre étude. L'objectif était de déterminer les facteurs liés au retard de négativation des BK dans les crachats. Il s'agissait d'une étude rétrospective analytique incluant 160 patients qui ont été suivis au service de pneumologie de l'Hôpital Fattouma Bourguiba de Monastir pour tuberculose pulmonaire confirmée de 2010 à 2020. La population d’étude était subdivisée en deux groupes selon le délai de négativation des BK dans les crachats relevés selon les dates des examens de laboratoires réalisés: 1^er^ groupe (G1) incluant des patients avec un délai de négativation des BK ≥ 2 mois; 2^e^ groupe (G2) incluant des patients avec un délai de négativation des BK < 2 mois. Nous avons colligé 160 patients d’âge moyen 44,2 ± 17,1 ans avec une prédominance masculine (119 hommes; 74,4%; sex-ratio = 2,9). Le 1^er^ groupe comportait 21 patients, soit 13,1%. Le retard de négativation des BK était plus fréquent chez les hommes (p = 0,05). Le tabagisme et l'alcoolisme étaient plus fréquents au sein du G1 avec des taux respectivement de 61,9% et 28,5% (avec p = 0,05 et p = 0,01 respectivement). Sur le plan radiologique, la présence d'un infiltrat micronodulaire à la TDM thoracique était associée à un retard de négativation. Cet aspect était présent chez 33% des sujets du G1 vs 14% du G2 (p = 0,05). La comparaison entre les deux groupes n'avait pas révélé une différence statistiquement significative en termes de comorbidités et d'observance. Plusieurs facteurs peuvent être associés au retard de négativation des BK, ce qui rend la maladie plus difficile à gérer. Une meilleure gestion de ces facteurs ainsi qu'une bonne éducation demeurent nécessaires afin de diminuer le délai de négativation des BK et donc limiter la propagation de la maladie.

# Research article: F045 - L'attitude des chirurgiens vis-à-vis des PvVIH

**Published:** 2023-10-05

**Authors:** F. Raqi, A. Oulad Lahsen, L. Marih, M. Sodqi, K. Marhoum El Filali

**Affiliations:** Service des maladies infectieuses, CHU Ibn Rochd, Casablanca, Maroc

Depuis le début de l’épidémie de VIH, les discriminations à l'encontre des personnes vivant avec le VIH (PvVIH) restent nombreuses. La stigmatisation joue un rôle non négligeable sur le développement et le maintien de l’épidémie de VIH. L'objectif est de décrire l'attitude des chirurgiens vis-à-vis des patients vivant avec le VIH afin de déceler des comportements discriminants, et de discuter l'intérêt d'une formation consacrée au VIH. Un questionnaire strictement anonyme et préalablement conçu depuis la plateforme Google Forms a été envoyé durant la période comprise entre le 30 novembre 2021 et le 13 septembre 2022 aux résidents chirurgiens du CHU Ibn Rochd de Casablanca. Un total de 83 résidents chirurgiens a été inclus dans l’étude dont 56,6% étaient de sexe masculin. Plus de 50% étaient âgés de moins de 30 ans, la majorité était en 5^e^ année de résidanat et 31,3% étaient des viscéralistes. 77,1% avaient déjà opéré des PvVIH, au moins 2 fois pour 67,6% des chirurgiens. 92,8% des participants n'avaient jamais refusé d'opérer les PvVIH dont 81,81% les considèrent comme les autres patients. Seulement 7,2% avaient refusé de les opérer malgré indication. 22,9% des chirurgiens n'ont jamais opéré des Pv-VIH dont la majorité (94,73%) était prête à les opérer si indication. L'impact négatif de la séropositivité VIH sur la qualité de la prise en charge a été rapporté par 31,3% des chirurgiens. Le besoin en formation sur le VIH a été exprimé par 91,4% des chirurgiens. La lutte contre la discrimination doit être incluse dans la stratégie de lutte contre la propagation de l'infection à VIH. L'une des solutions en milieu médical est le renforcement de la connaissance des personnels de santé en matière de VIH.

# Research article: F052 - Principaux effets indésirables des antifongiques: à propos de 36 cas

**Published:** 2023-10-05

**Authors:** H. Bouraoui, N. Gabsi, R. Laajimi, A. Chaabene, H. Ben Romdhane, N. Ben Fredj, N. Ben Fadhel, K. Aouam, Z. Chadli

**Affiliations:** Service de pharmacologie clinique, Faculté de médecine de Monastir, Maroc

Un antifongique est un médicament qui élimine sélectivement les champignons pathogènes avec une toxicité minimale pour l'hôte. De nombreux effets indésirables (EI) liés aux antifongiques, pouvant être parfois graves ont été reportés. L'objectif était de rapporter les différents EI liés à l'utilisation des antifongiques.

Nous avons mené une étude rétrospective incluant tous les patients ayant présenté des EI liés aux antifongiques notifiés au service de pharmacologie clinique de Monastir entre 2004 et 2022. L'imputabilité a été établie selon la méthode de Bégaud *et al.* Nous avons inclus 36 patients avec un sex-ratio H/F de 0,71. L’âge moyen était de 43,4 ans avec des extrêmes allant de 6 à 78 ans. Le délai moyen d'apparition était de 51 jours. Les antifongiques incriminés étaient: le fluconazole dans 15 cas, la terbinafine dans 8 cas, le voriconazole dans 6 cas, la griséofulvine dans 4 cas, l'amphotéricine B dans 2 cas et la combinaison de fluconazole et d'amphotéricine B dans 1 cas. Les EI notifiés étaient: une atteinte hépatique dans 17 cas (cytolyse dans 6 cas, atteinte mixte dans 4 cas, élévation isolée de GGT dans 3 cas, cholestase dans 2 cas et élévation isolée de la bilirubine dans 2 cas), un exanthème maculopapuleux (EMP) dans 6 cas, une photosensibilité dans 5 cas, un DRESS dans 2 cas, une pustulose exanthématique aiguë généralisée (PEAG) dans 1 cas, une pemphigoïde bulleuse dans 1 cas, un érythème pigmenté fixe (EPF) dans 1 cas, un urticaire dans 1 cas, une confusion dans 1 cas, et l'aggravation d'une insuffisance rénale dans 1 cas. Vingt-deux évènements ont été classés comme plausibles (I2), 8 comme vraisemblables (I3), et 6 comme douteux (I1). Des patchs tests ont été pratiqués chez 8 patients revenus négatifs. L’évolution était favorable pour tous les patients. À travers cette étude nous avons confirmé les données de la littérature concernant les principaux EI liés à la prescription de ces médicaments représentés essentiellement par les atteintes hépatiques, ce qui justifie la réalisation d'un bilan hépatique en pré-thérapeutique avec un contrôle toutes les 3-6 semaines après début du traitement.

# Research article: F054 - Critères épidémiocliniques d'orientation du diagnostic de leishmaniose cutanée

**Published:** 2023-10-05

**Authors:** F. Maatallah, N. Boulehmi, W. Aïssi, I. Ben Sghaier, O. Souissi, N. Zallagua, R. Ben Abdallah, E. Siala, A. Bouratbine, K. Aoun

**Affiliations:** Laboratoire de parasitologie-mycologie, LR 20-IPT-06, Institut Pasteur de Tunis

La leishmaniose cutanée (LC) est la maladie vectorielle la plus fréquente en Tunisie. Sa confirmation biologique est nécessaire avant l'administration de traitements contraignants, coûteux et parfois toxiques. L'objectif de ce travail est de dresser un profil des consultants pour suspicion de LC afin de proposer des critères d'orientation vers le diagnostic de la maladie. L’étude a concerné les patients adressés à l'Institut Pasteur de Tunis durant les années 2017 et 2018 pour confirmation biologique du diagnostic de LC. Une fiche anonyme a été remplie pour chaque patient relevant l’âge, le sexe, le lieu de contamination probable (déplacements), ainsi que le nombre, l'aspect morphologique, la localisation et l'ancienneté des lésions. Deux cent vingt-sept patients ont fait l'objet de l'analyse dont 101 (44,5%) ont été confirmés biologiquement (Microscopie et/ou PCR) et retenus cas de LC. Le sex-ratio H/F était de 1,02 avec un âge moyen de 34 ans chez les patients positifs contre 1,1 et 37,4 ans chez ceux négatifs. Les cas confirmés provenaient essentiellement du Centre et du Sud (n = 76 soit 75,2%). Le taux de positivité était supérieur chez les patients provenant du Centre et du Sud (61,3%) comparativement à ceux du Nord (24,5%). Les lésions ayant motivé la consultation étaient respectivement au nombre moyen de 2,9 chez les cas positifs et 2,2 chez les négatifs alors que leur ancienneté datait respectivement de 4,8 mois et de 6,3 mois. Les cas ont consulté pour 66,3% entre octobre et janvier contre 46% durant la même période chez les sujets négatifs. Le visage était touché à hauteur de 42,6% chez les cas et 35,7% chez les non-cas. Au-delà des résultats souhaités et indiscutables des examens parasitologiques, le profil des consultants suspects de LC peut s'avérer utile dans un cadre global de démarche diagnostique, particulièrement dans les zones pauvres en infrastructures de biologie médicale. Les sujets du Centre et du Sud consultant entre octobre et janvier et présentant plus de 2 lésions sembleraient plus à risque de LC.

# Research article: F056 - Étude épidémiologique et clinique de la leishmaniose cutanée dans la région de Sousse

**Published:** 2023-10-05

**Authors:** S. Mokni, M. Zorgati, S. Ismail, H. Chouaieb, I. Khammari, A. Yaacoub, A. Fathallah

**Affiliations:** Laboratoire de parasitologie-mycologie, Hôpital Farhat Hached, Sousse, Tunisie

La leishmaniose cutanée (LC) est une anthropozoonose très fréquente en Tunisie, représentant un problème de santé publique en raison de son incidence, sa morbidité et son coût élevé. L'objectif était de décrire les caractéristiques épidémiologiques et cliniques des cas de LC diagnostiqués au laboratoire de parasitologie-mycologie de l'hôpital Farhat Hached de Sousse. Il s'agissait d'une étude rétrospective de 3 ans (janvier 2020 – décembre 2022) portant sur tous les cas de LC diagnostiqués dans notre laboratoire. Les données ont été collectées à partir des fiches de renseignements réalisées systématiquement pour tous les patients adressés à notre laboratoire pour suspicion de LC. Le diagnostic a été fait par un examen direct (ED) des frottis dermiques colorés par MGG et/ou par PCR conventionnelle sur suc dermique. Durant la période d’étude, 615 demandes de diagnostic de LC ont été colligées. Le diagnostic était confirmé chez 256 patients (41,6%). L'ED était positif dans 253 cas. La PCR était positive chez 3 patients dont l'ED était négatif. L’âge moyen des patients atteints de LC était de 35,2 ans [3 mois – 97 ans], avec un sex-ratio (H/F) de 1,2. Les cas positifs étaient diagnostiqués surtout aux mois de décembre (74 cas) et novembre (53 cas). La notion de voyage dans une zone endémique était notée chez 186 patients (73,5%), principalement Kairouan (40%), Sidi Bouzid (32,4%) et Mahdia (16,2%). La notion de piqûre d'insecte était notée uniquement chez 51 patients (20,2%). Les localisations les plus fréquentes des lésions étaient celles des membres supérieurs (27,7%), membres inférieurs (24,9%) et visage (17,8%). Les lésions étaient multiples (≥ 2) dans 53% des cas. L'aspect ulcéro-croûteux était prédominant (74,3%). L’âge des lésions était supérieur à 1 mois dans 78,3% des cas, avec des extrêmes allant entre 1 semaine et 4 ans. La LC reste une pathologie endémique en Tunisie avec prédominance des formes multiples et ulcéro-croûteuses. La PCR est d'un grand apport en particulier devant une forte suspicion épidémiologique et clinique, et un ED négatif.

# Research article: F057 - Connaissances des militaires sur le paludisme

**Published:** 2023-10-05

**Authors:** A. Bouabdallah, S. Hannachi, N. Nabli, B. Nbiba, A. Bellili, R. Abid, R. Battikh

**Affiliations:** Service des maladies infectieuses, Hôpital militaire principal d'instruction de Tunis

Le paludisme est une maladie pouvant être grave et mortelle. Le personnel militaire est à risque de paludisme de par les missions dans les zones d'endémie palustre (ZEP). Nous avons étudié les connaissances de nos militaires tunisiens sur cette infection parasitaire. Nous avons réalisé un questionnaire que nous avons distribué à des militaires actifs. Nous avons colligé les réponses de 100 militaires (sex-ratio = 2,3). La tranche d’âge la plus représentée était celle des 31-40 ans (32%). Parmi eux, 39 ont déjà eu une mission à l’étranger, dont 30 en ZEP. Ces derniers ont reçu une formation sur le paludisme avant leur départ, que 9 ont jugée peu utile. Parmi les militaires interrogés, 96% avaient déjà entendu parler du paludisme, et 51% savaient qu'il était dû à un parasite. La contamination interhumaine était possible selon 49% des interrogés. La maladie pouvait être mortelle pour 84% d'entre eux. Trois quarts des militaires questionnés savaient qu'on pouvait contracter la maladie plusieurs fois, et 74% savaient que le paludisme n'existe plus en Tunisie. Concernant le mode de transmission, 29 cas l'ont rattaché à l'eau contaminée, 9 au lait non pasteurisé, et 90 à une piqûre d'insecte. Le symptôme le plus reconnu était la fièvre (98%) et le plus méconnu l'ictère (33%). Vingt-six personnes pensaient que le paludisme survenait au plus tard une semaine après le retour d'une ZEP. Quatre-vingt-six sujets savaient qu'il existe un traitement prophylactique et 23 qu'il faut le prendre même après le retour de la ZEP. Soixante-dix-sept militaires savaient qu'il existe un traitement curatif du paludisme. Aucun militaire n'avait répondu adéquatement à l'ensemble des questions portant sur les connaissances relatives au paludisme. Malgré les formations reçues, les connaissances des militaires sont insuffisantes en la matière. La réalisation systématique d'un frottis sanguin et d'une goutte épaisse au retour d'une ZEP chez les militaires et la bonne connaissance des médecins militaires de cette pathologie ont permis les prises en charge nécessaires à temps.

# Research article: F060 - Impact pronostic des endocardites infectieuses à staphylocoque

**Published:** 2023-10-05

**Authors:** Majed Hassine, Selma Kraeim, Kais Memmi, Marouen Mahjoub, Mehdi Boussadaa, Mejdi Ben Massoued, Fethi Betbout, Nidhal Bouchehda, Habib Gamra

**Affiliations:** Service de cardiologie A, Hôpital Fattouma Bourguiba, Monastir, Tunisie

Les endocardites infectieuses (EI) à staphylocoque prennent de plus en plus d'importance. Elles posent souvent aux cliniciens des problèmes pour la prise en charge et restent grevées d'un pronostic plus sombre. L'objectif était d’étudier les caractéristiques épidémiologiques et cliniques des patients hospitalisés pour EI à staphylocoque et d’évaluer le pronostic à court terme de ces patients. Un nombre total de 220 cas d'EI a été recensé dans notre registre durant une période étalée sur 13 ans entre janvier 2008 et décembre 2020. Nous n'avons inclus dans le groupe d’étude que les EI staphylococciques certaines. Le diagnostic d'EI à staphylocoque a été porté chez 53 patients (25%). L’âge moyen des patients du groupe EI à staphylocoque était de 36 ± 5 ans. L'atteinte valvulaire préexistante était la principale cardiopathie sous-jacente chez 18 patients (34%). L'EI à staphylocoque est survenue sur valve native chez 42 patients (79%). L'indication de chirurgie dans le groupe d'EI à staphylocoque a été portée chez 30 patients (57%). En comparant les principales complications survenues et le taux de mortalité dans notre groupe avec le groupe des EI non staphylococciques on retient que nos patients présentaient un pourcentage plus important d’état de choc (p = 0,047), d'insuffisance cardiaque (p = 0,001), d'insuffisance rénale (p = 0,001), de complications emboliques (p = 0,004), d'anévrisme mycotique (p = 0,001), d'abcès et de localisations septiques (p = 0,03) et un taux de mortalité plus élevé (37,7% vs 18%; p = 0,004). L'EI staphylococcique reste à l'origine d'une lourde morbi-mortalité et nécessite une stratégie d’évaluation accélérée qui offre la possibilité d'un diagnostic précoce et de décisions thérapeutiques appropriées chez des malades à risque plus important de complications et de décès.

# Research article: F063 - Pertinence de la prescription des carbapénèmes au cours des pyélonéphrites aiguës obstructives

**Published:** 2023-10-05

**Authors:** K. Chaker, Y. Ouanes, M. Trigui, M. Rinchi, B. Mosbahi, W. El Abed, O. Chbeb, H. Fakhfakh, M. Rahoui, M. Bibi, Kh. Mrad Dali, A. Ammous, Y. Nouira

**Affiliations:** 1 Service d'urologie, Hôpital La Rabta, Université de Tunis El Manar, Tunis, Tunisie; 2 Service d'anesthésie-réanimation, Hôpital La Rabta, Université de Tunis El Manar

## Introduction

La prescription des carbapénèmes (CBP) ne cesse de croître ces dernières années. Elle est expliquée par l’émergence d'entérobactéries productrices de bêta-lactamases à spectre étendu (BLSE). L'objectif de cette étude était d’évaluer la pertinence des prescriptions des carbapénèmes au cours des pyélonéphrites aiguës obstructives.

## Patients et méthodes

Nous avons mené une étude rétrospective colligeant tous les patients hospitalisés dans notre institution pour une pyélonéphrite aiguë obstructive entre janvier et décembre 2022. Nous avons évalué la prescription des carbapénèmes en nous basant sur l'index d'adéquation thérapeutique (IAT). Ce dernier repose sur un critère majeur qui est la conformité de l'indication par rapport aux recommandations de la Société de Pathologie Infectieuse de Langue Française (SPILF), et des critères secondaires qui sont la durée du traitement, la posologie, le choix d'une association antibiotique, la réévaluation à 48-72 h et l'adaptation de la prescription. La prescription était considérée comme non conforme lorsque le score IAT était supérieur à 3. Soixante-dix-neuf pyélonéphrites aiguës obstructives ont été traitées et drainées.

## Résultats

Un carbapénème a été prescrit dans 30 cas (39,24%). L’âge moyen des patients était de 52 ans. Le sex-ratio H/F était de 1,5. Les infections étaient nosocomiales dans 11 cas (13,92%). Une bactérie BLSE a été retrouvée dans l'ECBU vésical et/ou le prélèvement urinaire pyélique dans 26 cas (32,91%): *Escherichia coli* (n = 18), *Klebsiella pneumoniae* (n = 5), *Proteus mirabilis* (n = 3). La prescription des CBP était probabiliste dans 16 cas (dont 6 états de choc septique) et adaptée à l'antibiogramme dans 14 cas. L'imipénem était prescrit dans 28 cas et l'ertapénem dans 2 cas. La conformité était rapportée dans 25 cas (83,33%) pour l'indication d'un CBP, 25 cas pour la posologie (83,33%), 15 cas (50%) pour la durée, 13 cas pour le choix de l'association d'antibiotique (43,33%). L’évaluation globale trouve un IAT inférieur ou égal à 3 dans 24 cas (80%) traduisant la conformité de la prescription.

## Conclusion

Dans notre série, plus de 50% des pyélonéphrites aiguës obstructives ont nécessité la prescription de carbapénèmes. Cette prescription était non conforme aux recommandations dans le quart des cas.

# Research article: F074 - Les causes infectieuses de la fièvre prolongée inexpliquée: à propos de 116 cas

**Published:** 2023-10-05

**Authors:** M. Krifa, S. Kahloun, M. Garma, H. Boudrigua, M. Ben Ticha, F. Bellazreg, A. Letaief, W. Hachfi

**Affiliations:** Service des maladies infectieuses, CHU Farhat Hached, Sousse, Tunisie

## Introduction

Les principales causes de la fièvre prolongée inexpliquée (FPI) classique sont les maladies infectieuses, les maladies inflammatoires systémiques et les néoplasies. Près de 20% des cas de FPI demeurent sans cause identifiée. Le but de notre étude est de décrire les étiologies infectieuses de la FPI.

## Patients et méthodes

Il s'agit d'une étude transversale des patients hospitalisés pour FPI, entre janvier 2016 et mars 2021, aux services de maladies infectieuses et de médecine interne à l'hôpital Farhat Hached de Sousse. Une FPI était définie selon Durack et Street: fièvre ≥ 38,3 °C évoluant depuis plus de 3 semaines et dont le diagnostic n'a pas été établi après 3 jours d'hospitalisation ou au moins 3 consultations. Nous avons inclus 116 patients d’âge moyen 45 ans avec un sexratio de 1.

## Résultats

Une cause de la FPI était retenue chez 96 patients (82,8%) dont 70 cas (60,3%) étaient infectieux, 14 cas (12%) néoplasiques et 12 cas (10,4%) inflammatoires; chez 20 patients (17,2%) le diagnostic était resté indéterminé. Les pathologies bactériennes étaient majoritaires dans 65 cas (56%). Les infections à germe intracellulaire occupent la première place dans 26 cas (22,4%), suivies par l'endocardite 15 cas (12,8%), la tuberculose 8 cas (6,9%). Ces derniers étaient de localisation ganglionnaire (3 cas), hépatique (2 cas), pulmonaire (1 cas) et péritonéale (1 cas). Une double localisation hépatique et ganglionnaire était présente chez 1 patient. La brucellose concernait 6 cas (5%) dont 3 avaient une forme aiguë. Nous avons noté 2 cas d'EI brucellienne et 1 cas de spondylodiscite brucellienne. Chez 5 patients, l'infection était d'origine virale; primo-infection à CMV (2 cas); primo-infection à VIH (1 cas) et infection à virus West Nile (1 cas).

## Conclusion

Malgré les progrès de la recherche médicale, les étiologies infectieuses qui constituent une urgence diagnostique demeurent la première cause de la FPI. L’élaboration d'un algorithme de démarche diagnostique face à une FPI serait nécessaire afin de réduire le temps de délai diagnostique des pathologies infectieuses.

# Research article: F077 - La brucellose humaine dans la région de Sfax confirmée par l'isolement de la bactérie entre 2000 et 2022

**Published:** 2023-10-05

**Authors:** M. Guetat, N. Ben Ayed, F. Maalej, F. Smaoui, S. Mezghani, B. Mnif, F. Mahjoubi, M. Ben Jemaa, A. Hammami

**Affiliations:** 1 Laboratoire de microbiologie, CHU Habib Bourguiba, Sfax, Tunisie; 2 Service de maladies infectieuses, CHU Hédi Chaker, Sfax

## Introduction

La brucellose est une anthropozoonose qui sévit toujours à l’état endémique en Tunisie et pose donc un problème de santé publique. Une recrudescence a été observée au cours des dernières années. Le but du travail présenté était d’étudier le profil épidémio-clinique des brucelloses confirmées par culture bactérienne.

## Matériel et patients

Il s'agit d'une étude rétrospective s’étendant sur une période de 23 ans (2000 à 2022) et incluant toutes les souches de *Brucella* isolées au laboratoire de microbiologie de Sfax. L'identification du genre *Brucella* était basée sur les caractères morphologiques, culturaux et biochimiques. Pour tous les cas, la sérologie était pratiquée (séro-agglutination de Wright et test Rose Bengale).

## Résultats

Durant la période d’étude, 78 souches non répétitives de *Brucella* ont été colligées dont 38,6% ont été collectées les 5 dernières années. Les souches ont été isolées majoritairement à partir des hémocultures (81,3%) suivies par le LCS (11,3%). L’âge moyen des patients était de 42,6 ans (12 à 84 ans). Une prédominance masculine a été notée (sex-ratio = 1,85). Parmi les patients 37,7% étaient originaires de la région de Sfax, 33,3% de la région du Centre-Ouest (Sidi Bouzid, Kasserine et Kairouan) et 24,4% de la région du Sud-Ouest de la Tunisie (Gafsa, Kebilli). Parmi les facteurs de risque: la notion de consommation de lait cru était présente dans 33 cas et le contact avec les animaux dans 23 cas. Cliniquement, les localisations secondaires étaient essentiellement la spondylodiscite (14 cas), suivie par la méningite (8 cas) et la sacro-illite (3 cas). La sérologie était positive dans tous les cas. L'association thérapeutique doxycycline-rifampicine était la plus utilisée.

## Conclusion

L'incidence de la brucellose a connu une augmentation ces dernières années. Il convient donc de renforcer le contrôle de la brucellose animale afin d'empêcher l'apparition de nouveaux foyers de brucellose humaine.

# Research article: F078 - Particularités diagnostiques et thérapeutiques des rickettsioses dans un pays endémique

**Published:** 2023-10-05

**Authors:** Cyrine Kharrat, Salma Ben Hmida, Sirine Chtourou, Ichrak Bougharriou, Fatma Hammami, Fatma Frikha, Makram Koubaa, Chakib Marrakchi, Boussayma Hammami, Emna Elleuch, Mounir Ben Jmeaa

**Affiliations:** Service des maladies infectieuses, CHU Hédi Chaker, Sfax, Tunisie

## Introduction

Les rickettsioses sont des maladies d'inoculation dues à des bactéries intracellulaires strictes. La confirmation diagnostique est difficile en routine. L'objectif était de décrire les particularités diagnostiques et thérapeutiques des rickettsioses.

## Matériel et patients

L’étude présentée était rétrospective incluant les patients hospitalisés pour une rickettsiose durant 13 ans (2010-2023). Le diagnostic était retenu par la sérologie et/ou par la PCR.

## Résultats

Nous avons colligé 76 cas (45 femmes et 31 hommes) dont l’âge médian était de 42 ans [14-79]. Un contact avec les chiens était rapporté dans 58 cas (76,3%). Une escarre d'inoculation était trouvée dans 18 cas (23,7%). Soixante et un patients (80,3%) avaient une éruption cutanée. Le diagnostic microbiologique de la rickettsiose était fait par la sérologie dans 65 cas (85,5%) et/ou par la PCR (sur une biopsie cutanée ou sur un écouvillonnage) dans 11 cas (14,5%). Une infection à *Rickettsia conorii* était trouvée dans 68 cas (89,5%) et une infection à *Rickettsia typhi* dans 8 cas (10,5%). Cinquante-neuf patients (77,6%) étaient traités par doxycycline 200 mg/j pendant une durée moyenne de 6 [2-19] jours. Le délai moyen d'apyrexie sous traitement était de 3 [1-10] jours. L’évolution était favorable dans 75 cas (98,7%).

## Conclusion

Le tableau clinique des rickettsioses est polymorphe. La sérologie peut être négative dans les premiers stades de la maladie. Ainsi la PCR sur une biopsie cutanée et/ou sur un écouvillon par grattage représente une alternative dans le diagnostic microbiologique.

# Research article: F079 - Infections bactériennes et usagers de drogue intraveineuse: étude épidémioclinique, microbiologique et thérapeutique

**Published:** 2023-10-05

**Authors:** M. Maazoun, L. Ammari, B. Mahdi, A. Mbarek, O. Smaoui, R. Abdelmalek, A. Berriche, B. Kilani

**Affiliations:** Service des maladies infectieuses, Hôpital La Rabta, Tunis, Tunisie

## Introduction

L'usage de drogue intraveineuse (UDI) représente un fléau social de plus en plus répandu. Il expose également à un haut risque infectieux, notamment d'infections bactériennes.

## Patients et méthodes

L'objectif de ce travail est d’étudier les caractéristiques épidémiocliniques, microbiologiques et thérapeutiques des infections bactériennes chez les UDI. L’étude était rétrospective, menée au service des maladies infectieuses du CHU La Rabta à Tunis sur une période de 10 ans (janvier 2013 – décembre 2022) incluant tous les UDI hospitalisés pour une infection bactérienne.

## Résultats

Nous avons colligé 93 épisodes chez 76 UDI. Il s'agissait de 71 hommes (93,4%) et 5 femmes (6,6%). L’âge moyen était de 36,6 ± 8 ans. Le tabac (98,7%), l'alcool (75%), le comportement sexuel à risque (65,8%) et l'incarcération antérieure (39,5%) étaient rapportés. Les principales substances injectées étaient la buprénorphine (75%), la cocaïne (14%) et l'héroïne (3%). L’échange de seringue (64,5%) et l'utilisation d'eau du robinet (27,6%) étaient signalés. Une infection par le VHC (80,3%) ou le VIH (21,1%) était notée. Il s'agit de bactériémies (81,7%), d'infection des parties molles (11,8%), d'infections ostéo-articulaires (4,3%), d'infections du système nerveux central (2,2%). Les principales localisations secondaires des bactériémies étaient pulmonaires (68,4%), endocardiques (60,5%), ostéo-articulaires (30,3%), musculaires (21%) et spléniques (11,8%). La confirmation microbiologique était établie dans 66 épisodes (71%). Le germe isolé était *Staphylococcus aureus* (82%), *Staphylococcus* à coagulase négative (6%), ***Streptococcus*** (6%) et bacille à gram négative (BGN) (6%). Les souches de *Staphylococcus* isolées étaient sécrétrices de pénicillinase dans 24 cas (50%) et résistantes à la méticilline dans 10 cas (17,2%). Un seul BGN était multirésistant. Les associations d'antibiotiques les plus prescrites en empirique étaient oxacillinegentamicine (51,6%), céfazoline-gentamicine (17,2%) et glycopeptides-gentamicine (8,6%). Ce traitement s'est avéré adapté dans 83,9% des cas. Les antibiotiques de relais oral étaient pristinamycine (53,8%), fluoroquinolones (32,3%), rifampicine (22,6%) et cotrimoxazole (12,9%). La durée totale du traitement était de 44 ± 29 jours. L’évolution était favorable dans 76 épisodes (81,7%) avec 11 cas de décès (11,8%). Dix-sept épisodes de récidive notés étaient significativement associés à la mortalité (29,4% vs 7,9%; p = 0,026).

## Conclusion

L'UDI est un facteur de risque d'infection bactérienne. *Staphylococcus* représente le principal agent causal. Une prévention basée sur l'aide au sevrage est nécessaire pour éviter les récidives et diminuer la mortalité.

# Research article: F083 - Étude de la dynamique de circulation des virus respiratoires durant les années 2020/2021 et 2021/2022 de la pandémie Covid-19 en Tunisie

**Published:** 2023-10-05

**Authors:** Awatef Taktak, Saba Gargouri, Amel Chtourou, Mouna Maaloul, Fahmi Smaoui, Adnene Hammami, Héla Karray, Lamia Feki-Berrajah

**Affiliations:** Laboratoire de microbiologie, CHU Habib Bourguiba, Sfax, Tunisie

## Introduction

Depuis son émergence en décembre 2019, la circulation du SARS-CoV-2 a été associée à un changement du profil épidémiologique des autres virus respiratoires. Notre objectif était d’étudier la dynamique de circulation des virus respiratoires durant les années 2020-2021 et 2021-2022 de la pandémie Covid-19.

## Matériel et méthodes

Il s'agit d'une étude rétrospective menée au laboratoire de microbiologie du CHU Habib Bourguiba de Sfax. Ont été inclus les prélèvements nasopharyngés de 504 patients âgés de plus de 10 ans, chez qui une infection respiratoire aiguë était suspectée et testés négatifs pour le SARS-CoV-2. L’étude a comporté deux périodes: P1 (Oct 2020 – Mai 2021; 248 échantillons) et P2 (Sept 2021 – Mai 2022; 256 échantillons). Les virus respiratoires suivants ont été recherchés par RT-PCR en point final et PCR en temps réel: Virus Influenza (IFVA, IFVB et IFVC), Virus Respiratoire Syncytial, Métapneumovirus humain, les Virus Parainfluenza, Entérovirus/Rhinovirus humains (HEV/HRV), Adénovirus et Coronavirus classiques.

## Résultats

Le taux de positivité global pour au moins un seul virus a été de 27,8% et de 18,3% pour les périodes P1 et P2, respectivement. La plus faible circulation des virus respiratoires a été observée au mois de janvier 2022 (6,9%) coïncidant avec la flambée des contaminations par le VOC Omicron. HEV/HRV a été le virus le plus détecté représentant 61,9% et 58,4% des virus identifiés durant P1 et P2, respectivement. Durant P1, caractérisée par l’émergence de quelques variants du SARS-CoV-2 et l'application généralisée des mesures barrières, la détection des IFV a été très faible (2,8%). Cependant, à partir du mois de janvier 2022 (P2), une reprise de la circulation des IFV, représentés par le sous-type H3, a été observée (13,2%).

## Conclusion

La pandémie de Covid-19 a considérablement impacté la circulation des virus respiratoires notamment durant la saison 2021-2022, caractérisée par la flambée épidémique du VOC Omicron. Seule la circulation des HEV/HRV a été maintenue due à leur grande résistance dans l'environnement.

# Research article: F085 - Intérêt d'une technique de criblage « in house » pour la caractérisation des nouveaux sous-variants Omicron du SARSCoV-2

**Published:** 2023-10-05

**Authors:** W. Ayadi, S. Gargouri, A. Chtourou, F. Smaoui, A. Taktak, S. Ferjani, I. Boutiba, A. Hammami, H. Karray, L. Feki

**Affiliations:** 1 Laboratoire de biotechnologie moléculaire des eucaryotes, Centre de biotechnologie, Sfax, Tunisie; 2 Laboratoire de microbiologie, CHU Habib Bourguiba, Sfax; 3 Laboratoire de microbiologie, CHU Charles Nicolle, Tunis

## Introduction

À la suite de l’émergence de nouveaux sous-variants d'Omicron, beaucoup d'efforts ont été déployés afin de surveiller leur circulation dans le monde. Nous avons validé des outils de criblage conçus « in house » ciblant les variants BA.2, BA.4 et/ou BA.5 d'une part et BA.5, BQ.1, BA.2.75 et/ou XBB d'autre part.

## Matériels et méthodes

Il s'agit d'une étude rétrospective menée sur 189 prélèvements SARS-CoV-2 positifs collectés durant l’été 2022 (n = 154) et le mois de décembre 2022 (n = 35). Notre criblage repose sur la PCR allèle spécifique pour identifier la ΔHV69-70 du gène S et la ΔKSF141-143 du gène Orf1 et également la PCR-RFLP (412-476 aa; gène S) pour identifier les mutations K444T, V445P et L452R.

## Résultats

Notre analyse a montré, dans un premier temps, la ΔHV69-70 dans 143/154 cas (92,8%), suggérant ainsi une nette prédominance du BA.4 et/ou BA.5 durant l’été 2022 par rapport au sous-variant préexistant BA.2. La recherche de la ΔKSF141-143 a révélé, par la suite, le BA.5 dans 116 cas et le BA.4 dans seulement 27 cas. Quant à la 2^e^ période, décembre 2022, coïncidant avec l’émergence d'autres nouveaux sous-variants, la ΔHV69-70 était aussi observée dans la quasi-totalité des cas (32/35), suggérant ainsi la prédominance de BA.5 et/ou BQ.1 par rapport aux BA.2.75 et/ou XBB. La digestion par DdeI/MspI a montré un profil en faveur du BQ.1 dans 24 cas et du BA.5 dans 8 autres cas. Concernant les trois sous-variants non délétés HV69-70, le profil de digestion par BfaI était en faveur du BA.2.75 dans 2 cas et du XBB.1 dans le 3^e^ cas. Il est intéressant de noter que le séquençage du génome entier réalisé pour 94 parmi les 189 cas a confirmé nos résultats de criblage: BA.4 (n = 8), BA.5 (n = 59), BQ.1 (n = 24), BA.2.75 (n = 2) et XBB (n = 1).

## Conclusion

Cette étude souligne l'intérêt potentiel des tests de criblage rapide, comme technique complémentaire au séquençage, dans la stratégie de surveillance des sousvariants émergents du SARS-CoV-2.

# Research article: F087 - Place des herpèsvirus dans les infections neuroméningées dans la région de Sousse

**Published:** 2023-10-05

**Authors:** I. Handous, H. Kacem, Y. Maatouk, M. Marzouk, S. Ghorbel, H. Miladi, L. Bougamoura, N. Hannachi, J. Boukadida

**Affiliations:** 1 Laboratoire de microbiologie, CHU Farhat Hached, Sousse, Tunisie; 2 Service de pédiatrie, CHU Farhat Hached, Sousse

## Objectif

Les infections neuroméningées (INM) associées aux herpèsvirus (HHV) sont associées à une morbidité et une mortalité importante, tant chez les patients immunocompétents que chez les patients immunodéprimés. Le but de cette étude est d’étudier la prévalence de différents HHV dans les INM.

## Matériel et méthode

L’étude a concerné la recherche des HHV sur des échantillons de liquide cérébro-spinal (LCS) parvenus au laboratoire de microbiologie du CHU Farhat Hached de Sousse entre janvier et décembre 2019.

## Résultats

Les LCS avaient une pléiocytose lymphocytaire et une culture bactérienne négative. La recherche de virus a été faite par PCR multiplexe spécifique pour la détection des virus HSV-1/2, VZV, EBV, CMV, HHV-6 et HHV-7. Deux autres PCR ont été utilisées pour le typage de HSV1/2 et pour la quantification et le typage de HHV-6 (A/B). Les échantillons de LCS (n = 149) provenaient de patients âgés de 1 mois à 84 ans (médiane 30 ans). Une infection virale a été détectée dans 21,5% des échantillons (n = 32). Le virus le plus fréquemment identifié était le CMV (15,43% des cas). Le HHV-6 A/B était le deuxième virus détecté (3,34%,) suivi de HSV-1 et EBV (1,34% chacun) et du VZV (0,67%). Concernant les infections par HHV-6, il s'agissait de HHV-6A dans 2 cas et HHV-6B dans un autre cas, un patient était co-infecté par HHV-6A et HHV-6B et un autre co-infecté par CMV et HHV6B. La charge virale moyenne de HHV-6 sur LCS était de 15 680 UI/mL. Il n'y avait pas de lien entre âge et INM à HHV. Ces infections étaient significativement plus fréquentes chez les immunodéprimés que les immunocompétents (44,4% contre 16,3%, p = 0,003). La forme clinique d'INM la plus fréquemment associée à ces infections était la méningo-encéphalite (p = 0,01). Il n'a pas été noté d'association entre l'infection à HHV et l’évolution de la maladie vers le décès (p = 0,38).

## Conclusion

Les HHV sont des agents majeurs d'INM notamment chez l'immunodéprimé. La place de HHV6A/B paraît importante dans ces atteintes et sa recherche plus fréquente devant un tableau d'INM devrait être encouragée.

# Research article: F088 - Analyse moléculaire et durée d'excrétion virale du SARSCoV-2 chez les patients suivis pour hémopathies

**Published:** 2023-10-05

**Authors:** Y. Mhalla, A. Themlaoui, M. Hamdoun, H. Hannachi, M. Bahri, M. Sayadi, N. Jendoubi, D. Frioui, R. Belakhal, O. Bahri

**Affiliations:** Laboratoire de microbiologie, Hôpital Aziza Othmana, Tunis, Tunisie

## Objectif

Le Covid-19 peut être particulièrement grave chez les patients atteints d'hémopathies malignes. La gravité dépend du type de pathologie sous-jacente et du traitement immunosuppresseur. L'aspect virologique et son impact sur l’évolution de l'infection a été très peu étudié chez ces patients.

## Matériel et méthode

Étude descriptive du Covid-19 en hématologie qui a pour but d'analyser la corrélation clinico-virologique du Covid-19, d’évaluer la durée d'excrétion du SARS-CoV-2 et d'analyser la diversité génétique du virus. Étude rétrospective portant sur 37 patients suivis en hématologie à l'hôpital Aziza Othmana et infectés par le SARS-CoV-2.

## Résultats

Le diagnostic de Covid-19 a été confirmé par amplification du génome viral en temps réel (RT-PCR) sur prélèvements nasopharyngés. Des prélèvements réguliers ont été réalisés jusqu’à négativation de la RT-PCR afin d'estimer la durée d'excrétion virale. L'analyse moléculaire a été faite, en cas de RT-PCR (Ct). L’âge moyen des patients était de 37 ans avec prédominance féminine (n = 22; 62,2%). Les pathologies hématologiques étaient essentiellement la leucémie aiguë (n = 16; 43%) et le lymphome (n = 16; 43%). Dans 14% des cas, il s'agissait de LLC (n = 2), d'AHAI (n = 1) et de myélome (n = 1). Tous ces patients étaient en cours de traitement spécifique. L'infection à SARS-CoV-2 était asymptomatique dans 54% des cas (n = 20). Dans les formes symptomatiques, la fièvre et la toux sèche étaient les plus fréquentes (24% et 18% respectivement). Une évolution grave a été observée dans 5 cas dont 2 décès. La durée moyenne de l'excrétion virale était de 36 jours (de 7 à 92 jours); elle a dépassé les 30 jours dans 81% des cas (n = 30). Aucune différence significative n'a été notée selon le type de pathologie hématologique et selon le traitement en cours. Les différentes lignées virales identifiées étaient AY.122.6, B.1.1.7, BA.1.1, BA.1.1.1, BA.1.17.2, BA.2, BA.5.2, BA.5.2.1, BA.5.2.20 et BE.1; le variant omicron (lignée BA.1.1) a été le plus fréquent (n = 12; 32,4%). Au total, 8 (cas du variant Delta) à 37 mutations (variant Omicron) ont été retrouvées au niveau de la région S du génome viral. Deux mutations additionnelles ont été fréquemment retrouvées en cas d'infection par le variant Omicron; il s'agissait de Δ142 +Y145D qui ont été associées essentiellement à la résistance aux anticorps monoclonaux neutralisants spécifiques du domaine NTD de S1. Par ailleurs, une évolution génétique presque stable a été notée lors de l'analyse des souches retrouvées dans les prélèvements d'un même patient.

## Conclusion

Le Covid-19 est caractérisé par une excrétion virale prolongée en cas d'hémopathie maligne. Néanmoins, la réplication virale semble être associée à une diversité génétique stable; les principales mutations acquises affecteraient essentiellement l'infectiosité et la transmissibilité virale.

# Research article: F091 - Cinétique d'excrétion et caractérisation moléculaire des Entérovirus excrétés par des patients atteints d'immunodéficience primaire

**Published:** 2023-10-05

**Authors:** Imene Ben Salem, Haifa Khemiri, Nadia Driss, Najla Mekki, Imen Ben Mustapha, Henda Touzi, Zina Meddeb, Mehrez Yahyaoui, Mohamed Ridha Barbouche, Henda Triki, Sondes Haddad-Boubaker

**Affiliations:** Laboratoire de virologie clinique, Institut Pasteur de Tunis

## Objectif

Les patients à déficit immunitaire primaire (DIP) sont à haut risque pour contracter des infections virales récurrentes notamment par les Entérovirus poliomyélitiques (PV) et non poliomyélitiques (NPV). Ils présentent un potentiel accru d'excréter ces virus pendant de longues périodes, ce qui constitue une vraie menace pour la santé publique.

## Matériel et méthode

Dans ce travail, la cinétique de l'excrétion ainsi que la caractérisation moléculaire des souches excrétées ont été investiguées chez des patients atteints de DIP et excréteurs d'Entérovirus. L’étude a porté sur 247 prélèvements issus de 92 patients, originaires de 18 gouvernorats tunisiens. La recherche des EV a été réalisée par isolement sur culture cellulaire suivi d'un typage par des RT-PCR en temps réel. La caractérisation moléculaire a été réalisée par séquençage de la région VP1 et analyse phylogénétique.

## Résultats

Parmi les 92 patients investigués, huit étaient excréteurs d'Entérovirus (1 PV et 7 NPV) soit 8,69%. Le taux d'isolement des Entérovirus était de 5,6% majoritairement chez les patients atteints de DIP de type commun variable (CVID) (62%). L'excrétion s'est avérée de courte durée (1 à 5 mois) avec un faible taux d'excrétion de PV. La souche de PV excrétée était de type vaccinal 1 (SL1) et a présenté un taux de variabilité au niveau de la région VP1 de 0,9% indiquant une multiplication d'une durée de 11 mois. Cinq sérotypes d'NPV (E25, E19, E13, CVA5, CVB2) ont été identifiés dont l'analyse phylogénétique montre que ces souches sont majoritairement associées à des souches européennes et africaines.

## Conclusion

Ce travail contribue à une meilleure prise en charge des patients DIP.

# Research article: F093 - Incidence et facteurs prédictifs de la néphrotoxicité à l'aciclovir

**Published:** 2023-10-05

**Authors:** H. Knani, S. Rouis, M. Ben Selma, N. Ben Lasfar, M. Abid, M. Ben Ticha, F. Bellazreg, A. Letaief, W. Hachfi

**Affiliations:** 1 Service de maladies infectieuses, CHU Farhat Hached, Sousse, Tunisie; 2 Service de maladies infectieuses, CHU Ibn El Jazzar, Kairouan, Tunisie

## Objectif

L'aciclovir (ACV) est prescrit pour le traitement des infections sévères à Herpès simplex virus (HSV) et au virus de varicelle et du zona (VZV). Le traitement est habituellement bien toléré, mais il peut engendrer une insuffisance rénale aiguë (IRA) par précipitation des cristaux dans les tubules rénaux. Notre objectif était de déterminer l'incidence et les facteurs prédictifs de néphrotoxicité induite par l'ACV sur une période de 6 ans allant de 2017 à 2022, au service de maladies infectieuses de Sousse.

## Matériel et méthode

Les patients colligés étaient traités avec au moins une dose d'ACV, ayant une créatinine de base avec un contrôle régulier d'un jour sur trois au cours du traitement et après l'arrêt. L'IRA était définie selon les critères d'Acute Kidney Injury (AKIN) par une augmentation > 40 μmol/L par rapport à la valeur de base. Des analyses univariées et multivariées des facteurs de risque ont été réalisées pour identifier les facteurs prédictifs de néphrotoxicité de l'ACV. Au total 105 patients ont été inclus, avec une prédominance masculine (64,8%). L’âge médian était de 48 ± 19,7 ans.

## Résultats

Une infection à VZV était notée dans 48,57% des cas et l'infection à HSV dans 24,76% des cas. L'ACV était prescrit à la dose de 10 mg/kg/8 heures chez 96,2% des patients avec une durée moyenne de 7 ± 5,9 jours. Une antibiothérapie était associée à l'ACV dans 32,4%. Quarante-cinq patients (42,9%) n'avaient aucun antécédent pathologique, le diabète et l'hypertension artérielle étaient notés dans 14,3% des cas. Une infection au Virus d'immunodéficience humaine (VIH) a été notée dans 12,4% des cas. Le taux moyen de la créatinine de base était de 65 ± 37,6 pmol/L. Sept patients (6,7%) ont développé une IRA avec un âge médian de 67 ± 16,94 ans. Le délai médian d'apparition de l'IRA était de 6 ± 4,6 jours. La créatinine moyenne de contrôle chez ces patients était de 226 ± 119 μmol/L. Deux patients développant une insuffisance rénale étaient hypertendus et un patient était diabétique. L'analyse uni et multivariée de notre population d’étude n'a pas montré de facteurs indépendamment associés à la survenue de néphrotoxicité à l'ACV. L'absence de comorbidités était un facteur protecteur avec un risque relatif de 0,88 (0,80-0,96) (p = 0,017).

## Conclusion

Notre étude a montré que l'incidence de néphrotoxicité liée à l'ACV était de 6,7% avec un délai médian de 6 jours. L'absence de comorbidités était un facteur protecteur. De larges études prospectives et multicentriques s'avèrent nécessaires pour avoir de meilleurs résultats.

# Research article: F094 - Évaluation de la réponse immunitaire cellulaire au vaccin anti-SARS-CoV-2 chez le professionnel de la santé

**Published:** 2023-10-05

**Authors:** Y. Mhalla, B. El Kissi, A. Ben Salah, Y. Kadri, F. Slama, S. Mhalla, M. Mastouri

**Affiliations:** Laboratoire de microbiologie, CHU Fattouma Bourguiba, Monastir, Tunisie

## Objectif

La pandémie à SARS-CoV-2 qu'a connue le monde a particulièrement atteint, au début de sa généralisation, le personnel de la santé (PS) exposé aux malades. Dès le développement des vaccins, cette population a été prioritaire afin de garantir leur protection et par conséquent celle de leurs patients. Cependant, devant l'urgence de la situation la réponse vaccinale, aussi bien humorale neutralisante que cellulaire, n'a que très peu été étudiée. Notre objectif était d’étudier la réponse immunitaire à médiation cellulaire par dosage de l'interféron Gamma (IFN-γ) chez le PS ayant reçu une vaccination complète contre le SARS-CoV-2.

## Matériel et méthode

Une étude prospective comparative menée durant l'année 2022 sur des PS de notre CHU ayant ou non des antécédents d'infection par le SARS-CoV-2. La population a été divisée en deux groupes: le 1^er^ n'ayant jamais contracté le virus (groupe 1), et le 2^e^ ayant des antécédents de Covid (groupe 2). Des prélèvements sanguins ont été réalisés chez tous les sujets pour la détection qualitative de l'interféron-γ (IFN-γ) produit par les cellules T CD4+ et CD8+ et qui reflète la réponse immunitaire cellulaire, à l'aide du test QuantiFERON SARS-CoV-2 (Qiagen). Le recueil du consentement des sujets a été relevé *via* un formulaire clair. L'analyse statistique complète n'a pas pu être réalisée vu le faible échantillonnage.

## Résultats

Au total, 49 PS ont été inclus dont 31 n'avaient aucun antécédent de Covid (groupe 1) et 18 ayant déjà eu au moins un épisode de Covid (groupe 2). L’âge moyen était de 35 ans avec une prédominance féminine (65%, n = 32). Notre population a été composée de 21 médecins et 28 paramédicaux exerçant dans notre laboratoire ou au service des urgences. Aucun des PS ne prenait de traitement immunosuppresseur au cours de la période de l’étude et deux patients étaient asthmatiques sous traitement de fond. Tous les patients étaient vaccinés: 44% par un vaccin à ARN messager (Pfizer BioNTech 42% et Moderna 2%), 28% par un vaccin à vecteur viral (Spoutnik) suivi de rappel par Pfizer BioNTech, 8% par un vaccin à vecteur viral (6% Spoutnik, 2% Astrazeneca), 10% par un vaccin à virus inactivé type Sinovac et 4% type Sinopharm. Le résultat du test QuantiFERON était positif dans 71,4% (n = 35) dont 65% (n = 23) dans le groupe 1, avec une prévalence plus importante chez les sujets ayant reçu le vaccin (Pfizer BioNTech, n = 14) avec un schéma vaccinal de trois doses du vaccin. Le test était négatif dans 28,6% des cas (n = 14) dont 57% (n = 8) du groupe 1 et 43% du groupe 2 (n = 6). Parmi les sujets du groupe 1 IFN-γ, trois ont bénéficié d'une sérologie Covid qui a montré la présence d'anticorps neutralisants anti-SARS-CoV-2 mais qui ont développé un Covid dans les 2 mois qui ont suivi le test. D'après nos résultats, la réponse immunitaire cellulaire était plus importante chez le PS vacciné par Pfizer BioNTech ou après un Covid.

## Conclusion

Nous projetons de comparer la réponse cellulaire à celle humorale et d’étudier leur impact sur l'infection Covid. Ces données pourraient guider la prévention du virus dans la population générale.

# Research article: F095 - Analyse phylogénétique des lignées de SARS-CoV-2 en Tunisie entre mars 2020 et mars 2021

**Published:** 2023-10-05

**Authors:** S. Ferjani, I. Landolsi, M. Ben Sassi, I. Mkada, M. Arbi, M. Safer, A. Elmoussi, S. Abid, O. Souiai, S. Ayed, R. Daghfous, R. Gouider, J. Ben Khelil, M. Kharrat, I. Kacem, N. Ben Alya, A. Benkahla, S. Trabelsi, I. Boutiba-Ben Boubaker

**Affiliations:** 1 Laboratoire de microbiologie, Hôpital Charles Nicolle de Tunis, Laboratoire national de référence de la grippe et des autres virus respiratoires; 2 Université Tunis El Manar, Faculté de médecine de Tunis, LR99ES09; 3 Centre national Chalbi Belkahia de pharmacovigilance de Tunis; 4 Département de neurologie, LR18SP03, Hôpital Razi, Tunis; 5 Laboratoire de bio-informatique et biostatistique, LR16IPT09, Institut Pasteur de Tunis; 6 Observatoire national des maladies nouvelles et émergentes, Tunis; 7 Hôpital Abderrahmen Mami, Département de réanimation médicale, Ariana, Tunisie; 8 Université Tunis El Manar, Faculté de médecine de Tunis, LR99ES10 Génétique humaine

## Objectif

Depuis le début de la pandémie Covid-19, il y a eu un intérêt croissant pour l'exploration des variations génétiques de SARS-CoV-2 afin de comprendre l'origine et la propagation de la pandémie, d'améliorer les méthodes de diagnostic et de développer les vaccins appropriés. L'objectif de cette étude était d'identifier les lignées de SARS-CoV-2 circulant en Tunisie et ce afin de suivre leur transmission.

## Matériel et méthode

Au total, 90 souches de SARS-CoV-2 ont été incluses dans cette étude selon deux stratégies d’échantillonnage: échantillonnage aléatoire stratifié (n=32) et échantillonnage aléatoire simple (n=68). L'extraction de l'ARN viral a été réalisée à l'aide de l'extracteur automatique Perkin Elmer en utilisant le kit Chemagic 360. La détection du virus a été faite par RT-PCR en temps réel selon le protocole Hong Kong. Le séquençage du génome complet a été réalisé par le kit COVIDSeq.

## Résultats

De multiples introductions de lignées SARS-CoV-2 ont été notées au cours de la phase initiale de la pandémie, y compris B.4, B.1.1, B.1.428.2, B.1.540 et B.1.1.189. Par la suite, les lignées B1.160 (24,2%) et B1.177 (22,4%) ont été dominantes tout au long de l'année. Le variant Alpha (lignée B.1.1.7) a été identifié en février 2021 dans les régions du centre de notre pays. De plus, une nette diversité de lignées a été observée dans le Nord du pays. Ces résultats ont contribué à définir l’épidémie de Covid-19 en Tunisie.

## Conclusion

Malgré les ressources limitées du pays, la surveillance génomique du SARSCoV-2 doit être poursuivie pour détecter l’éventuelle émergence de nouveaux variants.

# Research article: F096 - La vague Delta en Tunisie: diversité génétique, distribution spatio-temporelle et évidence de la propagation d'une sous-lignée AY.122 divergente

**Published:** 2023-10-05

**Authors:** Sondes Haddad-Boubaker, Marwa Arbif, Oussema Souiaif, Anissa Chouikha, Wasfi Fares, Kate Edington, Sam Sims, Cesare Camma, Allessio Lorusso, Moussa Moïse Diagne, Amadou Diallo, Ilhem Boutiba Ben Boubaker, Sana Ferjani, Maha Mastouri, Salma Mhalla, Hela Karray, Saba Gargouri, Olfa Bahri, Abdelhalim Trabelsi, Ouafa Kallala, Naila Hannachi, Yassine Chaabouni, Hanen Smaoui, Khaoula Meftah, Sophia Besbes Bouhalila, Soumaya Foughali, Mariem Zribi, Asma Lamari, Henda Touzi, Mouna Safer, Nissaf Ben Alaya, Alia Ben Kahla, Mariem Gdoura, Henda Triki

**Affiliations:** 1 Laboratory of clinical virology, WHO Regional reference laboratory for poliomyelitis and measles for the EMR, Institut Pasteur de Tunis, University of Tunis El-Manar, Tunisia; 2 LR20IPT02, Laboratory of viruses, hosts and vectors, Institut Pasteur de Tunis, University of Tunis El Manar; 3 Clinical investigation center (CIC), Institut Pasteur de Tunis, Université Tunis El Manar; 4 LR16IPT09, Laboratory of bioinformatics, biomathematics and biostatistics, Institut Pasteur de Tunis, University of Tunis El-Manar; 5 New variant assessment platform (NVAP), Quadrum Institute, Norwich, United Kingdom; 6 Instituto zooprofilattico sperimentale dell'Abruzzo e del Molise G. Caporale (IZSAM), Teramo, Italy; 7 Department of virology, Pasteur Institute of Dakar, Dakar, Senegal; 8 Laboratory of microbiology, Charles Nicolle Hospital, Tunis, Tunisia; 9 LR99ES09, Laboratory research “Antimicrobial resistance”, Faculty of medicine of Tunis, University of Tunis El-Manar; 10 Laboratory of microbiology, Fattouma Bourguiba Hospital, Monastir, Tunisia; 11 LR99ES27, Laboratory research “Laboratoire des maladies transmissibles et substances biologiquement actives”, Faculty of pharmacy of Monastir, University of Monastir; 12 Laboratory of microbiology, Habib Bourguiba Hospital, Sfax, Tunisia; 13 Laboratory of microbiology and biochemistry, Aziza Othmana Hospital, Tunis; 14 Laboratory of virology, Sahloul Hospital of Sousse, Sousse, Tunisia; 15 Laboratory of microbiology, Farhat Hached Hospital of Sousse; 16 Laboratory of medical biology, Ibn El Jazzar Hospital, Kairouan, Tunisia; 17 Faculty of medicine of Tunis, University of Tunis El-Manar; 18 LR18ES39, Microbiology of children and immunocompromised, Faculty of medicine of Tunis, University of Tunis El-Manar; 19 Laboratory of microbiology, Bechir Hamza Children's Hospital, Bab-Saadoun Square, Tunis, Tunisia; 20 Laboratory ofmedical biology and blood bank, Institut Mohamed Kassab d'orthopédie, Manouba, Tunisia; 21 Laboratory of medical biology, Menzel Bourguiba Hospital, Bizerte, Tunisia; 22 Laboratory of microbiology, La Rabta Hospital, Tunis; 23 National observatory of new and emergent diseases, Tunis; 24 Faculty of pharmacy of Monastir, University of Monastir

## Objectif

Le variant Delta du virus SARSCoV-2 a posé un risque accru pour la santé publique mondiale et a rapidement remplacé les variants préexistants dans le monde. Dans cette étude, la diversité génétique et la dynamique spatio-temporelle de 662 génomes du variant Delta, obtenus à travers la Tunisie, ont été étudiées.

## Matériel et méthode

Le séquençage du génome viral entier et du segment partiel du gène S a été assuré au niveau de plateformes Illumina et Sanger, respectivement. L'attribution de la lignée a été réalisée par le logiciel Pangolin. Les séquences obtenues ont été investiguées par des analyses phylogénétique et phytogéographique.

## Résultats

La répartition par âge des cas infectés a montré un pic important entre 25 et 50 ans. Douze sous-lignées Delta ont été détectées à l’échelle nationale, AY.122 était le variant prédominant représentant 94,6% des séquences. Les séquences AY.122 étaient très apparentées génétiquement et partageaient le changement de l'acide aminé ORF1a:A498V et les mutations silencieuses 2746T>C, 3037C>T, 8986C>T, 11332A>G dans ORF1a et 23683C>T au niveau du gène S par rapport au Génome de référence de Wuhan (NC_045512.2). L'analyse phytogéographique indique que les grandes villes de Nabeul, Tunis et Kairouan ont constitué des épicentres pour la propagation de la souslignée AY.122 dans le reste du pays.

## Conclusion

Cette étude a permis d'enrichir les connaissances sur la distribution du variant Delta dans le monde en documentant les données tunisiennes, une région d'Afrique du Nord. De tels résultats seraient utiles pour la compréhension des futures vagues du Covid-19.

# Research article: F100 - Les infections fongiques invasives lors des leucémies aiguës myéloblastiques de l'adulte

**Published:** 2023-10-05

**Authors:** N. Siala, Y. Fakhfakh, N. Khemakhem, W. Feki, I. Ben Amor, H. Trabelsi, H. Fendri, O. Kassar, I. Frikha, F. Kallel, M. Charfi, L. Khelifa, R. Mallek, F. Makni, M. Medhaffar, A. Ayadi, Z. Menif, M. Elloumi

**Affiliations:** 1 Service d'hématologie clinique, CHU Hédi Chaker, Sfax, Tunisie; 2 Laboratoire de parasitologie, CHU Habib Bourguiba, Sfax; 3 Service de radiologie, CHU Hédi Chaker, Sfax

Les infections fongiques invasives (IFI) constituent une complication grave postchimiothérapie et sont associées à une morbi-mortalité élevée en onco-hématologie notamment lors des leucémies aiguës myéloïdes (LAM), alourdissant ainsi la prise en charge et le pronostic des patients.

## Objectif

Décrire les caractéristiques (épidémiologiques, cliniques, diagnostiques, thérapeutiques et évolutives) des IFI chez les adultes traités pour une LAM.

## Matériel et méthodes

Notre étude est rétrospective, menée au service d'hématologie clinique du CHU Hédi Chaker de Sfax, Tunisie, sur une période de 15 ans (janvier 2005 – décembre 2019), concernant les cas de LAM de l'adulte jeune (20-60 ans) traités par CT et dont l’évolution était compliquée par la survenue d'une IFI. Le diagnostic des IFI repose sur un faisceau d'arguments cliniques, radiologiques, microbiologiques et anatomopathologiques selon les critères d'ECIL.

## Résultats

Cent vingt-trois épisodes d'IFI ont été enregistrés chez 102 cas parmi les 171 LAM traités (60%). L’âge médian était de 42 ans et le sex-ratio était de 1,31. Les facteurs de risque des IFI étaient: le tabagisme dans 27 cas, le diabète dans 5 cas et des antécédents respiratoires dans 2 cas. La prescription d'antibiothérapie à large spectre, la neutropénie profonde et prolongée, et l'absence de prophylaxie antifongique étaient notées dans tous les cas (100%). Les épisodes IFI étaient diagnostiqués durant le traitement d'induction dans 92 cas et lors des cures de consolidation dans 31 cas. Nous avons noté 88 cas d'aspergillose, 27 cas de candidose, 5 cas de septicémie à *Geotricum capitatum,* 2 cas de septicémie à *Trichosporon asahi* et 1 cas de mucormycose. Les IFI étaient associées dans 21 cas: une aspergillose associée à une candidose dans 16 cas, une aspergillose associée à une septicémie à *G. capitatum* dans 3 cas, une candidose associée à une septicémie à *T. asahi* dans 1 cas et une candidose associée à une septicémie à *G. capitatum* dans 1 cas. Le traitement comprenait 91 monothérapies [voriconazole (n = 75), fluconazole (n = 8), caspofongine (n = 5), amphotéricine B (n = 3)] et 11 bithérapies. L’évolution était favorable pour 80 patients alors que 22 patients sont décédés suite à l'IFI (par état de choc septique dans 10 cas, pneumopathie hypoxémiante dans 8 cas et hémorragie intra-alvéolaire secondaire à l'extension de lésion d'aspergillose dans 4 cas).

## Conclusion

La fréquence d'IFI dans notre série de LAM est supérieure à celles rapportées (60% vs 7-12%). L'installation de systèmes de filtration de l'air ainsi que l'instauration d'un traitement antifongique prophylactique pourrait améliorer nos résultats.

# Research article: F101 - Le complexe *Trichophyton mentagrophytes :* étude épidémiologique et moléculaire

**Published:** 2023-10-05

**Authors:** N. Khemakhem, S. Neji, H. Trabelsi, H. Sellami, F. Makni, A. Ayadi

**Affiliations:** Laboratoire de parasitologie-mycologie, CHU Habib Bourguiba, Sfax, Tunisie

*Trichophyton mentagrophytes* est un dermatophyte cosmopolite. Sa fréquence est en nette recrudescence dans notre région. C'est un complexe formé par des espèces anthropophiles et d'autres zoophiles. Il présente une variabilité phénotypique importante et l'identification correcte des espèces reste encore problématique.

## Objectif

Étudier le profil épidémiologique des dermatophyties à *T. mentagrophytes* et analyser la variabilité génotypique de certaines souches par PCR-RFLP et PCR-séquençage.

## Matériel et méthodes

Étude rétrospective réalisée sur 65 059 patients suspects de dermatomycoses adressés à notre laboratoire durant une période de 21 ans (1999-2019). L’étude moléculaire a été réalisée pour 61 souches de *T. mentagrophytes.* Nous avons amplifié les régions ITS par les amorces ITS1 et ITS4. Les enzymes utilisées pour la digestion enzymatique étaient MvaI et HinfI.

## Résultats

36 278 (55,76%) patients présentaient une dermatophytie. *T. mentagrophytes* a été isolé chez 1671 patients (4,6%). L’âge moyen était de 39,7 ans. Le sex-ratio était de 1,1. La forme clinique la plus fréquente était la dermatophytie plantaire (29,8%) suivie par les onyxis (25,5%) et les intertrigos (24%). La PCR-séquençage a confirmé l'identification morphologique de *T. mentagrophytes* dans 93,44% des cas et a redressé le diagnostic dans 4 cas *(T. rubrum).* L'alignement multiple de nos séquences a montré un pourcentage de similarité de 100% avec les séquences de *T. interdigitale.* La PCR-RFLP a généré un seul profil alors que l'analyse des séquences ITS a distingué 4 génotypes. Le génotype dominant était de type I caractérisant la variante anthropophile de *T. interdigitale.* La PCR-séquençage a aussi identifié *T. mentagrophytes* var. *erinacei* isolé d'une dermatophytie des mains pour la première fois dans notre pays. La PCR-RFLP était incapable de détecter le polymorphisme intraspécifique des souches du complexe *T. mentagrophytes.* Par ailleurs, la PCR-séquençage était utile pour l'identification correcte et la distinction des espèces anthropophiles et zoophiles de ce complexe. Cette différenciation est pertinente car elle permet de cerner l'origine de la contamination et de prescrire le traitement adéquat.

# Research article: F102 - Identification moléculaire et test de sensibilité *in vitro* aux antifongiques des souches *d'Aspergillus* section *Nigri* isolées de cas d'otomycose en Tunisie

**Published:** 2023-10-05

**Authors:** H. Elkamel, S. Belgacem, M. Lakoudi, Z. Jlassi, M. Mastouri

**Affiliations:** Laboratoire de parasitologie-mycologie, CHU Fattouma Bourguiba, Monastir, Tunisie

*Aspergillus niger* est le principal agent responsable d'otomycose dans le monde. Ce champignon fait partie *d'Aspergillus* section *Nigri,* un ensemble d'espèces morphologiquement indiscernables qui peuvent héberger divers modèles de sensibilité aux antifongiques.

## Objectif

Le but de cette étude était d'identifier et de déterminer avec précision le schéma de sensibilité d'un ensemble d'aspergillus noirs isolés chez des patients atteints d'otomycose.

## Matériel et méthodes

Étude rétrospective allant du 1^er^ janvier 2019 au 31 décembre 2020 portant sur tous les examens mycologiques auriculaires positifs à *Aspergillus* noirs, identifiés microscopiquement et par séquençage partiel du gène codant pour la bêta-tubuline. La sensibilité des isolats à la caspofungine (CAS), l'anidulafungine (AND), l'amphotéricine B (AMB), l'itraconazole (ITR), le voriconazole (VOR), le posaconazole (POS), le kétoconazole (KET) et la flucytosine (5FC), a été testée en suivant la procédure M38-A2 du Clinical and Laboratory Standards Institute (CLSI).

## Résultats

26 souches *d'Aspergillus* noirs ont été isolées. L’âge médian des patients était de 39 ans [20-73 ans], la tranche d’âge la plus touchée était entre 20 et 40 ans, le sex-ratio était égal à 0,53. Ces otites aspergillaires ont été diagnostiquées dans un tiers des cas pendant l'hiver, l'examen direct était positif dans seulement 46% des cas montrant des filaments mycéliens. Ces souches ont été identifiées comme *A. niger* selon les méthodes morphologiques classiques alors que l'analyse moléculaire a permis d'identifier 3 souches d’*Aspergillus* appartenant à la section *Nigri* **:** *A. tubingensis* 46% (n = 12), *A. niger* et *A. welwitschiae* (27%) pour chacun. L’étude de la sensibilité aux antifongiques a montré que la totalité des souches avaient une concentration minimale effective inférieure ou égale à 0,25 μg/mL aux deux échinocandines testées. Les valeurs de concentration minimale inhibitrice (CMI) les plus basses ont été observées pour le VOR et POS [0,015-1 pg/mL], concernant le kétoconazole, 19 souches avaient une valeur de CMI supérieure ou égale à 8 pg/mL, 69% des souches d’*Aspergillus* testées avaient une CMI inférieure ou égale à 4 pg/mL pour l'itraconazole, 85% des souches avaient une valeur de CMI inférieure ou égale à 2 pg/mL à l'amphotéricine B, la 5FC a montré des valeurs de CMI élevées [2-32 pg/mL].

## Conclusion

Notre étude indique que *A. tubingensis* était environ deux fois plus répandue que *A. niger* chez les patients atteints d'otomycose. Cependant, ces données proviennent d'un seul centre et ne peuvent pas être étendues à d'autres régions de la Tunisie.

# Research article: F104 - Identification moléculaire et sensibilité aux antifongiques des espèces *d'Aspergillus* responsables d'otomycose à Monastir

**Published:** 2023-10-05

**Authors:** S. Belgacem, H. Elkamel, W. Marrakchi, M. Lakoudi, Z. Jelassi, S. Miled, M. Mastouri, H. Babba

**Affiliations:** 1 Laboratoire de microbiologie, CHU Fattouma Bourguiba, Monastir, Tunisie; 2 Laboratoire de recherche de parasitologie-mycologie, Faculté de pharmacie, Monastir; 3 Service des maladies infectieuses, CHU Fattouma Bourguiba, Monastir

L'otomycose est le plus souvent bénigne, mais la symptomatologie occasionnée, la résistance fréquente aux traitements, et les récidives multiples en font une source de frustration pour le patient et pour le clinicien.

## Objectif

Le but de cette étude était de déterminer le spectre des espèces d’*Aspergillus* responsables d'otomycoses, de comparer la caractérisation phénotypique et moléculaire et d’étudier le profil de leurs sensibilités aux antifongiques.

## Matériel et méthodes

Étude rétrospective allant du 1^er^ j anvier 2019 au 31 décembre 2020, portant sur tous les examens mycologiques auriculaires positifs à *Aspergillus.* L'identification phénotypique était basée sur les caractéristiques microscopiques et macroscopiques. La caractérisation moléculaire était réalisée grâce au séquençage du gène codant pour la bêta-tubuline. Pour l’étude de la sensibilité aux antifongiques, on a appliqué les recommandations du Clinical and Laboratory Standards Institute en suivant la procédure M38-A2, en testant huit molécules, par la technique de micro-dilution en milieu liquide. L'analyse des résultats a été réalisée par le logiciel SPSS.

## Résultats

68 souches d’*Aspergillus* spp. ont été isolées, avec une prévalence de 40%. L’âge moyen des patients était 50 ans. Le sex-ratio était égal à 0,48. Nos résultats ont montré une correspondance entre l'identification phénotypique et l'identification moléculaire égale à 69%. Le séquençage moléculaire a montré que 38% des souches d’*Aspergillus* appartenaient à la section *Nigri,* 44% faisaient partie de la section *Flavi* et 4% étaient classées dans la section *Terrei*. L'analyse du gène codant pour la bêta-tubuline a permis l'identification de nouvelles souches cryptiques telles *qu'Aspergillus citrinoterreus, Aspergillus tamarii, Aspergillus oryzae, Aspergillus tubingensis* et *Aspergillus welwitschiae.* L’étude de la sensibilité aux antifongiques a montré que la totalité des souches aspergillaires isolées avaient une concentration minimale efficace inférieure ou égale à 0,25 pg/mL pour la caspofungine, et 0,125 pg/mL pour l'anidulafungine. Plus de la moitié des souches avaient une valeur de concentration minimale inhibitrice (CMI) supérieure ou égale à 0,5 pg/mL pour le voriconazole. Pour le posaconazole, 76% des isolats de la section *Flavi* avaient une CMI inférieure ou égale à 0,25 pg/mL et seulement un cinquième des souches appartenant à la section *Nigri* avaient une CMI inférieure ou égale à 0,25 pg/mL. Concernant l'itraconazole, 85% des souches de la section *nigri* avaient une valeur de CMI supérieure ou égale à 4 pg/mL, quant à la section *flavi,* les trois quarts des isolats avaient une valeur de CMI égale à 1 pg/mL. La majorité des souches d’*Aspergillus* testées à l'amphotéricine B avait une valeur de CMI supérieure ou égale à 2 pg/mL.

## Conclusion

Afin d’élaborer des recommandations thérapeutiques nationales pour la prise en charge des otomycoses aspergillaires, des études supplémentaires avec un échantillonnage plus grand seront nécessaires pour identifier le spectre des espèces d’*Aspergillus* et surveiller la résistance aux antifongiques.

# Research article: F0106 - Diagnostic anténatal de la toxoplasmose congénitale: l'indication est-elle toujours justifiée?

**Published:** 2023-10-05

**Authors:** R. Trabelsi, R. Ben Abdallah, O. Souissi, R. Maatoug, K. Aoun, A. Bouratbine

**Affiliations:** Laboratoire de parasitologie-mycologie, Institut Pasteur de Tunis

Le diagnostic anténatal de la toxoplasmose (TC) est souvent réalisé devant une infection toxoplasmique pergravidique grâce à la PCR réalisée sur liquide amniotique (LA) à la recherche de l'ADN parasitaire. L'objectif de notre travail était de rapporter l'expérience de l'Institut Pasteur de Tunis (IPT) concernant l'indication du diagnostic anténatal de la TC.

## Matériel et méthodes

Étude rétrospective colligée au laboratoire de parasitologie-mycologie de l'IPT sur une période de 17 ans. Nous avons inclus les femmes enceintes adressées à notre laboratoire pour qPCR sur LA. Pour chaque femme, une fiche de renseignements a été établie. Pour les femmes qui n'ont pas été suivies dans notre laboratoire, nous nous sommes basés sur les sérologies faites dans d'autres laboratoires pour retenir un statut sérologique. Les échantillons de LA (10-20 mL) ont été prélevés par amniocentèse sous échoguidage par les gynécologues et acheminés directement au laboratoire.

## Résultats

Durant les 17 ans de l’étude, nous avons reçu 402 femmes enceintes pour qPCR, dont 11 n'avaient pas de critères d'infection toxoplasmique récente. La plupart de ces femmes (6/11) ont été adressées par des gynécologues de libre pratique. Toutes ces femmes ont eu leur première sérologie toxoplasmique avant 12 SA avec une moyenne de 9,6 ± 2,1 SA et des extrêmes allant de 6 à 12 SA, avec comme résultat, présence d'IgG et d'IgM anti-toxoplasmiques associée à un indice d'avidité des IgG élevé (ancienneté de plus de 20 semaines de la contamination). Huit femmes parmi les 11 ont été mises sous traitement préventif à base de spiramycine dès la première sérologie et l'amniocentèse a été pratiquée avant 18 SA chez 3 femmes.

## Conclusion

L'amniocentèse est une procédure médicale invasive utilisée pour le diagnostic anténatal de la TC et dont l'indication doit toujours être bien justifiée.

# Research article: F109 - Le parasitisme intestinal chez les étudiants: dépistage systématique toujours d'actualité

**Published:** 2023-10-05

**Authors:** J. Ben Jannet, S. Marouen, A. Kallel, A. Dalleli, S. Cheikhrouhou, B. Hmissi, M. Messaoud, S. Belhadj, K. Kallel

**Affiliations:** Laboratoire de parasitologie-mycologie, CHU La Rabta, Tunis

Dans le cadre du programme national de surveillance des étudiants non-résidents permanents en Tunisie (ENRPT), un examen parasitologique des selles est réalisé afin de dépister une éventuelle parasitose intestinale et la traiter.

## Objectif

Étudier la fréquence des parasitoses intestinales chez les ENRPT et identifier les différentes espèces parasitaires rencontrées.

## Matériel et méthodes

Étude descriptive rétrospective réalisée au laboratoire de parasitologie-mycologie du CHU La Rabta de Tunis durant une période de 26 mois [1^er^ janvier 2021 – 28 février 2023]. Nous avons colligé les ENRPT adressés par la direction de médecine scolaire et universitaire dans le cadre du programme national de surveillance de cette population. Pour chaque étudiant, nous avons réalisé un interrogatoire en remplissant une fiche de renseignements, un examen parasitologique des selles (examen direct, technique de concentration de Faust et technique de coloration MIF) et une coproculture parasitologique. Nous avons utilisé Microsoft Excel et SPSS comme outils statistiques.

## Résultats

Notre étude a porté sur 527 ENRPT. Le sex-ratio était de 1,94. La moyenne d’âge était de 25,4 ans [17-59 ans]. La majorité des ENRPT étaient originaires d'Afrique subsaharienne (66,5%), 18,2% d'Afrique du Nord et 15,3% d'Asie. Cliniquement, tous les étudiants étaient asymptomatiques. Sur les 527 ENRPT, 174 étaient parasités, soit une fréquence globale de 33%. La majorité des étudiants parasités étaient originaires d'Afrique subsaharienne (155 étudiants) soit 89% des étudiants parasités. La totalité des parasites mis en évidence étaient des protozoaires. *Entamoeba coli* était le parasite le plus fréquemment isolé, identifié chez 69 étudiants, soit 39,6% des parasités. Les parasites pathogènes rencontrés dans notre série étaient présents chez 54 étudiants, soit 31% des parasités et 10,2% des ENRPT. Le pathogène le plus rencontré était *Entamoeba histolytica* / *dispar* (n = 27) suivi de *Giardia intestinalis* (n = 19) et de *Dientamoeba fragilis* (n = 16).

## Conclusion

Les parasitoses intestinales sont encore répandues chez les ENRPT. Certains parasites pathogènes comme *E. histolytica* peuvent représenter un risque pour la population autochtone. De ce fait, une surveillance parasitologique systématique des ENRPT dans le cadre du programme national demeure nécessaire.

# Research article: F110 - Sécurité transfusionnelle et paludisme: les limites du dépistage sérologique

**Published:** 2023-10-05

**Authors:** E. Siala, G. Lakhdhar, A. Ben Khlil, I. Bouhaouala, H. Fares, N. Boulehmi, A. Bouratbine, K. Aoun

**Affiliations:** 1 Service de parasitologie-mycologie, Institut Pasteur de Tunis, Tunisie; 2 Laboratoire d’épidémiologie et d’écologie parasitaire, Institut Pasteur de Tunis

En sécurité transfusionnelle, la détection des porteurs de *Plasmodium* parmi les voyageurs et les immigrants provenant des zones d'endémie palustre se base sur le dépistage sérologique chez les donneurs de retour depuis plus de 4 mois.

## Objectif

Le but de notre étude était d’évaluer les performances diagnostiques d'un kit ELISA dans la détection des Ac *anti-Plasmodium*.

## Matériel et méthodes

Étude prospective concernant 56 individus adressés au service de parasitologie de l'Institut Pasteur de Tunis entre 2019 et 2022: 36 étudiants asymptomatiques originaires de zones d'endémie et ayant quitté leurs pays depuis plus de 4 mois et dont l'examen microscopique du sang était positif à *Plasmodium falciparum* dans 35 cas et à *Plasmodium ovale* dans 1 cas; 20 Tunisiens qui n'ont jamais voyagé en zones d'endémie palustre. L'examen microscopique du sang pour ces derniers était négatif. Tous ces individus ont bénéficié d'une détection des Ac sériques *anti-Plasmodium* par le kit *Anti-Plasmodium* ELISA (Euroimmun AG, Allemagne).

## Résultats

Quatre cas de sérologie négative ont été notés chez les 36 sujets asymptomatiques et porteurs de *Plasmodium*, alors que toutes les sérologies étaient négatives pour les 20 Tunisiens. La sensibilité du kit ELISA était de 89% et sa spécificité de 100%. Ses valeurs prédictives positives et négatives étaient respectivement de 100% et de 83%.

## Conclusion

Le dépistage sérologique du paludisme se heurte actuellement à une sensibilité insuffisante des techniques commercialisées. Par conséquent, l'amélioration de ces tests ou l'introduction de moyens plus performants comme la biologie moléculaire s'avère indispensable pour la sécurité transfusionnelle vis-à-vis de cette infection.

# Research article: F113 - Leishmaniose cutanée dans la région de Sfax, un motif fréquent de consultation

**Published:** 2023-10-05

**Authors:** S. Farhat, S. Louati, F. Cheikhrouhou, A. Kanoun, N. Khemakhem, F. Makni, A. Ayedi

**Affiliations:** Laboratoire de parasitologie-mycologie, CHU Habib Bourguiba, Sfax, Tunisie

La leishmaniose cutanée (LC) est endémique en Tunisie et demeure un réel problème de santé publique, malgré les efforts de lutte et de prévention.

## Objectif

Dans ce travail, nous décrivons les caractéristiques épidémiologiques et cliniques de la LC dans la région de Sfax.

## Matériel et méthodes

Il s'agissait d'une étude rétrospective portant sur 4728 patients de 2004 à 2022. Le diagnostic était confirmé par un examen direct des frottis dermiques après coloration Giemsa et/ou PCR/RFLP du suc dermique.

## Résultats

Le diagnostic de la LC a été confirmé pour 2877 cas. L’âge moyen des patients était de 31,9 ans (1 mois – 98 ans) avec un sexratio de 0,95. Deux pics ont été observés en 2015 (535 cas) et 2019 (605 cas). La majorité des cas étaient originaires de Sfax (71%). Les lésions étaient multiples (≥ 2) dans 66,8% des cas, et siégeaient majoritairement au niveau des membres inférieurs (39,5%) et supérieurs (35,4%), puis au niveau de la face (22,7%) et du tronc (4,1%). Pour la localisation faciale, on trouve en premier lieu les joues (38,3%) puis le nez (28%), le front (20,3%) et les oreilles (4,2%). L'aspect clinique des lésions était ulcéro-croûteux dans 66,8% des cas, avec la présence des formes sèches dans 18% des cas, ulcéreuses dans 12%, œdémateuses dans 5,4% et squameuses dans 4% des cas. Le diagnostic a été redressé par la PCR dans 447 cas. L'identification de l'espèce a été réalisée dans 207 cas: 201 *L. major* et 6 *L. killicki.*

## Conclusion

La LC est endémique, mais elle tend à s’étendre à d'autres régions, probablement en rapport avec les modifications climatiques, la désertification et les mouvements de populations.

# Research article: F117 - Place du Western Blot et de l'immunochromatographie dans la détermination du statut sérologique toxoplasmique chez la femme enceinte

**Published:** 2023-10-05

**Authors:** Y. Kalboussi, R. Ben Abdallah, R. Maatoug, O. Souissi, K. Aoun, A. Bouratbine

**Affiliations:** Laboratoire de parasitologie-mycologie, Institut Pasteur de Tunis

L'interprétation de la sérologie toxoplasmique chez la femme enceinte nécessite parfois le recours à des techniques complémentaires coûteuses telles que le Western Blot Toxo II IgG. Certains auteurs rapportent des performances similaires d'un test immunochromatographique rapide, l'ICT Toxo IgG-IgM.

## Objectif

Nous proposons dans ce travail d’évaluer les performances de ce test rapide par rapport au Toxo II IgG considéré comme le *gold standard* dans la détection des IgG dans certaines situations particulières.

## Matériels et méthodes

Il s'agissait d'une étude descriptive rétrospective menée au laboratoire de parasitologie-mycologie de l'Institut Pasteur de Tunis incluant toutes les femmes adressées pour le dépistage systématique de la toxoplasmose de 2015 à 2018. La recherche des anticorps anti-toxoplasmiques a été réalisée par ELISA. L’évaluation du test rapide a d'abord été faite sur tous les sérums ayant nécessité le recours à la technique du Toxo II IgG, puis ces sérums ont été répartis en trois groupes: sérums ayant un titre des IgG entre 6 et 9 UI/mL et IgM absentes; sérums ayant un titre des IgG entre 4,4 et 6 UI/mL et IgM absentes; sérums ayant un titre des IgG inférieur.

## Résultats

Nous avons inclus 39 femmes enceintes parmi 2615 adressées à notre laboratoire durant la période d’étude. L’étude comparative des résultats des deux techniques obtenus sur l’échantillon complet a conclu à une sensibilité de 100%, une spécificité de 73,3%, une valeur prédictive négative de 100%. La valeur prédictive négative ainsi que la sensibilité étaient de 100% dans les trois groupes. En revanche, la spécificité était de 50% à 69,3% dans le 2^e^ et le 3^e^ groupe, respectivement.

## Conclusion

Notre travail a pu conclure que le test immunochromatographique ICT Toxo IgG-IgM pourrait être utilisé comme technique de confirmation, permettant, s'il est négatif, d’éviter le passage au Western Blot Toxo II IgG. Par contre, en cas de positivité, ce dernier s'avère incontournable.

# Research article: F119 - Trichostrongylose humaine: premier cas en Tunisie diagnostiqué au laboratoire de parasitologiemycologie de l'hôpital Charles Nicolle

**Published:** 2023-10-05

**Authors:** F. Zaabi, D. Aloui, R. Ghodbane, M. Bouchekoua, S. Trabelsi

**Affiliations:** Laboratoire de parasitologie-mycologie, Hôpital Charles Nicolle, Tunis, Tunisie

## Introduction

Les nématodes du genre *Trichostrongylus* sont principalement des parasites des herbivores. Des cas d'infections humaines ont été rapportés dans de nombreux pays (Iran, Extrême-Orient, Australie…). En Tunisie, aucun cas n'a été décrit et cette infection est à ce jour méconnue.

## Objectif

Nous rapportons le premier cas d'une infection humaine par *Trichostrongylus* sp. diagnostiquée au Laboratoire de parasitologie-mycologie de l'hôpital Charles Nicolle de Tunis.

## Observation

Il s'agissait d'une fillette de 3 ans consultant au service de pédiatrie de l'hôpital Charles Nicolle de Tunis, pour anorexie, amaigrissement et retard de croissance. Sur le plan biologique, elle a présenté une anémie associée à une hyperéosinophilie. À l'interrogatoire, la patiente était originaire d'une zone rurale de Kasserine et vivant à Tunis. Elle était en contact avec des oiseaux, des volailles et des moutons. Trois examens parasitologiques des selles (EPS) ont été demandés, objectivant la présence d’œufs de *Trichostrongylus* sp. sur deux échantillons.

Une enquête de dépistage a été menée chez la famille, associant un interrogatoire minutieux et des EPS chez les parents qui n'avaient aucun facteur de risque de la trichostrongylose. Leurs EPS étaient négatifs.

La patiente a été traitée par une dose unique d'albendazole suivie de 3 jours de mébendazole. Le contrôle parasitologique n'a pas été fait (malade perdue de vue).

## Discussion

L'infection humaine se produit lorsque les larves sont ingérées avec des légumes à feuilles contaminés par de la terre contenant des excréments d'animaux herbivores. La plupart des infections sont asymptomatiques. Les infections graves peuvent provoquer des signes gastro-intestinaux (douleurs abdominales, diarrhée, anorexie), des maux de tête, une anémie et une hyperéosinophilie.

L'identification de ce parasite dans les selles est difficile en raison de la ressemblance morphologique de ses œufs avec ceux des ankylostomes.

## Conclusion

Les infections humaines par les strongles des animaux sont rares, expliquant la méconnaissance de la trichostrongylose humaine qui est certainement sous-estimée. La prévention repose sur le respect des règles hygiéno-diététiques qu'il faut promouvoir notamment chez les enfants.

# Research article: F120 - Immunogénicité de primeboost hétérologue et homologue avec un vaccin à virus non actif et à ARNm Covid-19: essai clinique phase III randomisé

**Published:** 2023-10-05

**Authors:** S. Samoud, AA. Gereisha, J. Bettaieb, M. Gdoura, G. Kharroubi, F. Ben Ghachem, I. Zamali, A. Ben Hamid, N. Hogga, M. Gharbi, C. Khemissi, Y. Galai, H. Louzir, H. Triki, M. Ben Ahmed

**Affiliations:** 1 Service d'immunologie clinique, Institut Pasteur de Tunis, Tunisie; 2 Faculté de médecine de Sousse, Université de Sousse, Tunisie; 3 Service d’épidémiologie médicale, Institut Pasteur de Tunis; 4 Faculté de médecine de Tunis, Université de Tunis; 5 Laboratoire de virologie clinique, Institut Pasteur de Tunis; 6 Faculté de pharmacie, Université de Monastir, Tunisie; 7 Centre de vaccination de la ville de l'Ariana, Direction régionale de la santé de l'Ariana, Ariana, Tunisie

La pandémie du SARS-CoV-2 constitue un problème de santé publique national et mondial. En un temps record, différentes plateformes vaccinales développées pour la plupart sur la base du schéma homologue ont été disponibles. Cependant, des études de qualité ont montré la supériorité du protocole de vaccination « prime-boost hétérologue » vaccin à ARNm/vaccin à adénovirus par rapport au protocole homologue.

Dans un essai clinique pionnier, nous avons comparé l'immunogénicité de deux doses de vaccins à virus inactivé CoronaVac/CoronaVac à celle d'une première dose de virus inactivé suivie d'un rappel par le vaccin à ARNm CoronaVac/BNT162b2. Nous avons évalué l'immunogénicité et la sécurité de ce schéma vaccinal afin de réviser et ajuster les recommandations nationales en matière de vaccination contre le Covid-19.

Il s'agit d'un essai clinique de supériorité randomisé en simple aveugle visant à mesurer les titres des anticorps neutralisants et les anticorps anti-Spike au niveau des prélèvements sanguins des participants qui ont reçu une première dose de CoronaVac suivie d'une dose de BNT162b2 ou de CoronaVac. Les participants étaient tous des adultes consentants, sains, sans notion d'infection antérieure au Covid-19. Le critère de jugement principal était le titre d'anticorps neutralisants avec un pourcentage d'inhibition à 90% 3 à 5 semaines après la deuxième dose vaccinale. Une différence de 25% entre les deux groupes était jugée significative.

Parmi les 240 participants, nous avons analysé le profil clinico-biologique de 100 participants recevant le schéma vaccinal hétérologue *versus* 99 recevant le schéma vaccinal homologue. La répartition des participants était randomisée. Les participants recevant le schéma vaccinal hétérologue ont présenté un titre significativement plus important d'anticorps neutralisants (médiane à 94%, intervalles interquartiles (IIQ) [81-96] *versus* 96% IIQ [95-97]) comparativement au groupe homologue (p < 0,0001). Par ailleurs, le pourcentage d'individus présentant un niveau d'anticorps neutralisants > 90% était plus important dans le groupe vaccinal hétérologue (90%) *versus* l'homologue (61%) (p < 0,0001). Aucun effet indésirable grave n'a été noté à 30 jours de la deuxième dose vaccinale chez les deux groupes. Cette étude montre la supériorité de l'immunogénicité du schéma vaccinal hétérologue CoronaVac/BNT162b2 comparativement au schéma homologue CoronaVac/CoronaVac.

# Research article: F122 - Conception d'une protéine vaccinale contre les variants de SARS-CoV-2 circulant durant l'hiver 2022-2023 par des approches immuno-informatiques

**Published:** 2023-10-05

**Authors:** F. Smaoui, S. Gargouri, A. Chtourou, M. Maaloul, A. Taktak, F. Frikha, A. Hammami, H. Karray-Hakim, A. Rebai, L. Feki-Berrajah

**Affiliations:** 1 Laboratoire de microbiologie, CHU Habib Bourguiba, Sfax, Tunisie; 2 Laboratoire des procédés de criblage moléculaire et cellulaire, Centre de biotechnologie, Sfax

Les vaccins anti-SARS-CoV-2 actuels s'appuient sur la protéine Spike. Néanmoins, celle-ci subit plusieurs mutations avec l’évolution des variants, diminuant l'efficacité des vaccins. Une solution est d'induire une réponse immunitaire focalisée sur les épitopes conservés qui pourrait offrir une protection prolongée.

## Objectif

Notre objectif était de concevoir une protéine vaccinale contre les principaux variants circulant durant l'hiver 2022-2023, combinant les épitopes conservés dans les domaines RBD et NTD de la protéine Spike.

## Matériels et méthodes

Les épitopes conservés qui sont reconnus par les lymphocytes B, T-CD4+ et T-CD8+ ont été prédits à travers la plateforme Immune Epitope Database Analysis Resource (IEDB). Ensuite, différents outils bio-informatiques (Vaxijen, Allertop”, Toxinpred”, etc.) ont été utilisés pour réaliser des étapes de sélection selon plusieurs critères: antigénicité, immunogénicité, allergénicité, auto-immunogénicité, toxicité, induction de cytokines.

## Résultat

Les peptides retenus ont été assemblés avec un adjuvant pour construire une protéine multi-épitope. Cette dernière a été évaluée en étudiant ses propriétés physicochimiques par le serveur PsiPred et en réalisant des simulations immunitaires et de docking. Au total, 16 épitopes ayant les propriétés souhaitables ont été retenus aux étapes de sélection. La proportion de la population mondiale ayant des allèles CMH-I et/ou II qui reconnaissent au moins un épitope T a été estimée à 98,68%. Après assemblage, une protéine vaccinale de 29,9 kDA comportant 281 acides aminés a été obtenue. La caractérisation physicochimique a montré sa stabilité (index d'instabilité = 28,72), sa thermostabilité (index aliphatique = 91,81), sa solubilité (score = 0,622) et sa durabilité dans l'organisme (demi-vie > 30 h dans les réticulocytes), tandis que les simulations ont prédit sa capacité à se fixer sur le TLR4 et à déclencher une réponse immunitaire adaptée.

## Conclusion

La protéine multi-épitope ainsi conçue semble être efficace et prometteuse pour protéger contre plusieurs variants. Des études *in vitro* et *in vivo* seront nécessaires pour confirmer ces résultats.

# Research article: F123 - Covid-19 chez le personnel de santé: cinétique de la réponse immunitaire humorale postvaccinale

**Published:** 2023-10-05

**Authors:** C. Naffouti, H. Hannachi, F. Ben Salem, I. Ayari, M. Hamdoun, S. Mechmech, M. Chouchene, O. Bahri

**Affiliations:** 1 Laboratoire de microbiologie-biochimie, Hôpital Aziza Othmana, Tunis, Tunisie; 2 Unité de médecine du travail, Hôpital Aziza Othmana, Tunis

Le Covid-19 représente un défi mondial. Différents types de vaccins ont été rapidement développés, mais leur efficacité serait compromise du fait de la diminution dans le temps de l'immunité acquise.

## Objectif

L'objectif de ce travail était d’étudier la cinétique des anticorps (Ac) obtenus après vaccination chez le personnel de santé (PS) considéré groupe à haut risque, et d'identifier les facteurs influençant cette cinétique.

## Matériels et méthodes

Étude longitudinale prospective descriptive menée entre mars 2021 et juin 2022 à l'hôpital Aziza Othmana. Elle a inclus tout le PS ayant été vacciné contre le Covid. Une recherche des Ac anti-S par chimiluminescence (ACCESS2-BECKMAN COULTER, États-Unis) a été réalisée avant la primo-vaccination (M0) pour définir le statut immunitaire initial puis à 1 et à 6 mois (M1 et M6). Une sérologie était considérée comme positive si le titre d'Ac était supérieur à 10 UA/mL (UA = Unité arbitraire).

## Résultats

Au total, 341 PS ont été inclus; l’âge moyen était de 41 ans [23-69] et le sexratio de 0,2. Le taux des séronégatifs était de 19%. Une réponse post-vaccinale a été observée dans 92% et 64,5% à M1 et M6 respectivement. Une baisse statistiquement significative des taux médians des Ac a été notée entre M1 et M6 allant de 91,8 UA/mL à 15 UA/mL (p = 0,004). Les vaccins à ARNm seraient plus efficaces; le titre des anticorps induits dans ce cas était statistiquement plus élevé à M1 et M6 par rapport aux vaccins à vecteur viral et les vaccins à virus inactivés (p = 0,0001). Le taux médian d'Ac à M1 était significativement plus élevé chez les sujets de sexe féminin que ceux de sexe masculin (136,6 UA/mL et 108 UA/mL respectivement; p = 0,019). Il était également statistiquement plus élevé en cas d'infection antérieure par SARS-CoV-2: 135,4 UA/mL *vs* 102,7 UA/mL chez les non infectés (p = 0,004).

## Conclusion

L'immunité humorale anti-SARS-CoV-2 n'est pas constante, elle baisse de façon significative à 6 mois après la primovaccination. Elle est également dépendante du type de vaccin administré. Des doses de rappel sont fortement recommandées, surtout en cas de vaccin à type de vecteur viral ou de vaccin inactivé.
